# Positive behavioural support for children and young people with developmental disabilities in special education settings: A systematic review

**DOI:** 10.1111/jar.12989

**Published:** 2022-02-23

**Authors:** Lorena Beqiraj, Louise D. Denne, Richard P. Hastings, Andreas Paris

**Affiliations:** ^1^ Centre for Educational Development, Appraisal and Research (CEDAR) University of Warwick Coventry UK; ^2^ Centre for Developmental Psychiatry and Psychology Monash University Clayton Victoria Australia

**Keywords:** autism, functional behaviour assessment, intellectual disability, positive behavioural support(s), special education, systematic review

## Abstract

**Background:**

Positive behavioural support (PBS) can be effective in supporting children and young people (CYP) with developmental disabilities. This systematic review focused on describing the components and nine characteristics of PBS that have been used with CYP with developmental disabilities in special education settings, and the evidence for PBS effectiveness in these settings. Additionally, facilitators and barriers to PBS implementation, and experiences of stakeholders, were investigated.

**Method:**

Systematic searches followed a registered protocol, and 30 studies were identified, narratively synthesised, and critically appraised.

**Results:**

From the 30 studies included, 10 reported the presence of all 9 PBS characteristics, 17 reported on 8 PBS characteristics, and 3 reported on 7 characteristics. Overall, 28 studies demonstrated significant decreases in behaviours that challenge and increases in alternative behaviours, if increasing alternative behaviours was part of the interventions.

**Conclusions:**

There was a lack of evidence on facilitators and barriers, and a lack of qualitative studies exploring experiences of stakeholders with PBS in special education settings. The available evidence suggested that not all studies reported on all PBS characteristics when describing the approach followed. In addition, available evidence suggested that most studies demonstrated effectiveness of PBS regarding the measured outcomes. Implications and future directions are discussed.

## 1 INTRODUCTION

Positive behavioural support (PBS)^1^ is defined as a multicomponent and multi‐tiered framework (Dunlap & Carr, 2007; Gore et al., 2013; Kincaid, 2018). The PBS framework focuses on person‐centred support developed with the involvement of stakeholders. People with developmental disabilities are at risk of developing behaviours that challenge (Hastings et al., 2013). The term neurodevelopmental disorders, otherwise referred to as developmental disabilities, is defined by the Diagnostic and Statistical Manual of Mental Disorders (5th ed.; DSM‐5; American Psychiatric Association, 2013) and the Centers for Disease Control and Prevention (CDC) (Centers for Disease Control and Prevention, n.d.) as a group of conditions that can cause impairments in learning, social and occupational functioning, and can also cause physical impairments. The term includes a wide range of conditions such as intellectual disability, autism, specific learning disorder, attention deficit/hyperactivity disorder (ADHD), and many more. The term developmental disabilities will be used in the text to refer to the specific conditions of intellectual disability (or genetic syndromes predominantly manifesting with an intellectual disability) and autism, that belong to the general category of developmental disabilities. Children and young people (CYP)^2^ with comorbid conditions of which at least one is either intellectual disability (or genetic syndrome causing an intellectual disability) or autism, will also be referred to as CYP with developmental disabilities. However, it is acknowledged that the term ‘developmental disabilities’ is commonly used to refer to the above‐mentioned conditions (i.e., intellectual disability, genetic syndrome causing an intellectual disability, autism) as well as other conditions (e.g., including ADHD). PBS places emphasis on understanding behaviour that challenges at a functional level, using that understanding to develop systems of support that increase the likelihood of preventing or reducing behaviour that challenges, and increasing the quality of life for the person and their family (Carr et al., 2002; Gore et al., 2013). Definitions of PBS reflect the different contexts in which the framework has been applied, including mainstream/inclusive schools in the USA and residential or community settings in the United Kingdom (Gore et al., 2013; Kincaid et al., 2016; Sugai & Horner, 2009), but also include three components: values, systems change, and the underpinning science and technologies.

The development of PBS was motivated by movements advocating equality for people with developmental disabilities, protection of their human rights (Gore et al., 2013), and their valued role in society (Wolfensberger, 2011). There are three dimensions to the values component, and these remain at the core of PBS: a constructional approach, a non‐aversive approach, and stakeholder involvement. PBS, as a proactive and constructional approach (Kincaid et al., 2016), focuses on expanding repertories of social and other functional competencies by building new skills, as well as redesigning environments to increase life opportunities for people in need of support (Gore et al., 2013; Kincaid, 2018). Behaviours that challenge may still occur (Carr et al., 2002), but skills‐building and increased life opportunities are a focus in their own right. PBS is guided by the philosophy of non‐aversive, respectful practices, and advocates for the use of alternatives to punitive technologies (Carr et al., 2002). Stakeholder participation is also an integral part of the PBS approach (Carr et al., 2002) and is incorporated at every step of the PBS implementation process, to ensure social validity and adherence to the supports provided.

The systems change component of PBS relates to the establishment of a ‘continuum of positive behaviour support’ (Lewis et al., 2002, p. 181) with interventions applied within a multi‐tiered framework at both the individual and a larger systems level (such as family and school contexts; Kincaid et al., 2016). A three‐tiered model for prevention and intervention informs supports progressively more individualised according to need, allowing organisations to scale‐up the support provided whilst achieving consistency across settings, support providers, and time.

The science and technologies component of PBS is underpinned by behaviour analytic principles, combined with other evidence‐based practices (Gore et al., 2013; Kincaid et al., 2016). Thus, PBS utilises knowledge derived from behavioural science and other scientific fields (such as pedagogical and implementation science) to develop an understanding that all behaviours have a function, what that function is, and how to design and scale‐up implementation of supports. These scientific principles define the technologies that are then applied to achieve socially valid outcomes. A strong theoretical and evidence‐based understanding of the function of behaviours that challenge is a primary characteristic of PBS (Hastings et al., 2013; Iwata et al., 1994). Functional behaviour assessment (FBA)^3^ procedures are designed to identify the function of behaviour and interventions informed by FBA are more effective than those not function‐informed (Ingram et al., 2005). PBS also utilises a data‐informed approach (Carr et al., 2002) to guide decision making (Horner & Sugai, 2018; Kincaid et al., 2016). Using data to make critical decisions about established supports minimises bias in intervention decisions and reduces influence of personal perspectives (Gore et al., 2013). Therefore, decision making is evidence‐informed by quantitative and qualitative data collected, aiming to avoid unsubstantiated personal assumptions.

PBS is not a single intervention or programme. It is rather a framework that incorporates a strong values base, systems change, and supports utilising scientific knowledge and technologies based on primarily, but not limited to, behaviour analytic principles. Behaviour analytic principles, deriving from behavioural psychology, underlie applied intervention programmes such as applied behaviour analysis (ABA) interventions as well. However, from the available evidence‐based interventions and supports only those relevant to individual needs and adhering to the core values of PBS are selected as appropriate for implementation within a PBS framework. PBS places emphasis on offering non‐aversive, respectful, and socially valid supports, that are proactive and constructional, to increase the quality of life of CYP (Carr et al., 2002;Gore et al., 2013; Kincaid et al., 2016). Therefore, only supports guided by these values are incorporated as part of the PBS framework. Moreover, PBS also includes the use of other evidence‐based practices, and incorporates systems change to scale‐up and achieve consistency of supports (Gore et al., 2013; Kincaid et al., 2016), making it distinct from single intervention programmes.

School‐wide positive behavioural support (SW‐PBS) is a successful preventative model in mainstream schools for all children, including those with special educational needs (Horner et al., 2009). A three‐tier model of prevention has been adopted, incorporating universal supports across the whole educational setting at the primary level (Tier 1), targeted supports for selected at risk groups at the secondary level (Tier 2), and specialist supports for CYP that require individualised support at the tertiary level (Tier 3; Sugai & Horner, 2006). However, PBS (in its individualised, group level, or school‐wide form—following the SW‐PBS model) has been implemented in special education settings internationally as well, in support of CYP with developmental disabilities. Moreover, there is also a research base evaluating PBS in alternative and special education settings (e.g., Clarke & Duda, 2019; Jolivette et al., 2012; Paris et al., 2019; Simonsen et al., 2010; Simonsen & Sugai, 2013; Wienen et al., 2019).

To date, systematic reviews on PBS and its effectiveness have either focused on PBS implementation to support CYP with disabilities in various settings, including PBS implementation in non‐educational settings (Snell et al., 2005), aggregate reporting of studies conducted in both mainstream and special educational settings (Goh & Bambara, 2012; Noltemeyer et al., 2019), reporting of the implementation of one specific tier of support, such as Tier 1, in alternative settings (including juvenile justice facilities; Grasley‐Boy et al., 2020), or have focused on effectiveness of function‐based PBS for CYP diagnosed with specific conditions such as emotional and behavioural disorders (Lane et al., 2009). Some of these systematic reviews included data on the effectiveness of PBS in special education settings as part of the aggregate reporting of studies conducted in various settings for CYP with various diagnoses. However, we were unable to find reviews that were conducted in special education settings addressing the implementation of PBS for CYP with developmental disabilities. The current systematic review focused on special education settings because it aims to describe the PBS framework characteristics, including essential adaptations made to achieve contextual fit for this specific setting, when supporting the target population. It also explores the effectiveness of these characteristics. This is particularly important considering the increasing number worldwide of special educational settings that choose to implement the PBS framework to support CYP with developmental disabilities, a group at risk for behaviours that challenge. This systematic review focused on the following questions:

*Review Question 1 (RQ1)*: How has PBS been implemented for CYP with developmental disabilities in special education settings, and what is the evidence on the effectiveness of PBS in improving outcomes for CYP with developmental disabilities in special education settings?

*Review Question 2 (RQ2)*: What are the perceived facilitators and barriers to implementation of a PBS approach with CYP with developmental disabilities in special education settings?

*Review Question 3 (RQ3)*: What are the experiences and views of stakeholders (CYP with developmental disabilities, parents/carers, school staff, other professionals, policy makers) about PBS interventions in special education settings?

## 2 METHODS

For the systematic review, the preferred reporting items for systematic reviews and meta‐analyses (PRISMA) guidelines (Page et al., 2021) were followed. The protocol was registered with the international prospective register of systematic reviews (PROSPERO 2019: CRD42019131954).

### 2.1 Search strategy

Seven electronic bibliographic databases were searched (PsycINFO, ERIC, MEDLINE, Applied Social Sciences Index and Abstracts (ASSIA), Social Science Citation Index (SSCI), Web of Science, and Scopus). The search strings included terms related to developmental disability (a): Intellectual Disability (e.g., Learning Disab*, Intellectual* Impair*, Down* syndrome) and autism (e.g., Autis* Spectrum Disorder*, Asperger* syndrome); (b): behaviour that challenges (e.g., Challenging behav*, Aggressive behav*); and (c): special education settings (e.g., Special educat* provision*, Alternat* educat* setting*, Resource* room). Terms within the same search group were separated by OR, and then combined using AND. An example search string can be found in the Table S1.

Backward and forward reference searches were also used. The forward reference search was conducted using the Social Sciences Citation Index (SSCI) and Google Scholar databases to identify citations of included studies. Specialists in the field of PBS in special education settings were also contacted and asked for copies of recent relevant research that had not yet been published. No copies of relevant research were provided, and therefore none were included in this systematic review. Given that the *International Journal of Positive Behavioural Support* (IJPBS) was not included in the major databases, hand searching of the IJPBS was conducted.

### 2.2 Inclusion criteria for studies

Empirical studies were eligible for inclusion if they met the inclusion criteria relating to Population, Intervention, Comparator, Outcomes, Study design, and Setting (PICOSS):

1. This systematic review focused on the implementation of the PBS framework to support specifically CYP with either intellectual disability, or autism, or both, exploring adaptations of the framework for these CYP groups. CYP with multiple diagnoses were eligible if they had received at least one of the above diagnoses. CYP with intellectual disability (including genetic syndromes such as Down's Syndrome or Angelman Syndrome, that predominately manifest with a comorbid intellectual disability) and/or autism who were aged 3–25 years old (or this age group consisted at least 70% of the sample, or data were reported separately for this age group) were included if: (i) they had a formal diagnosis of an intellectual disability (or genetic syndrome) and/or autism according to DSM‐5, ICD‐11, or equivalent, or (ii) they were administratively defined as having intellectual disability by being enrolled in a special education setting (e.g., special school), or in a special education provision that exists within a mainstream school. Studies were included if they reported data separately for CYP with intellectual disability and/or autism, or if they did not report data separately for them but this group was at least 70% of the research sample. In the latter case, a cut‐off of 70% was selected to ensure that any aggregate data included, reflected the majority of data from CYP with developmental disabilities.
2. The approach being implemented included the three components of PBS (Table 1). This multicomponent framework was developed to provide a defining and synthesis framework for the current review, and it was informed by an analysis of existing PBS definitions and the components and characteristics of the PBS approach (Allen et al., 2005, 2013; Carr et al., 1999, 2002; Dunlap et al., 2009; Gore et al., 2013; Kincaid, 2018; Kincaid et al., 2016; LaVigna & Willis, 2005; Morris & Horner, 2016). Papers describing characteristics of PBS to ensure contextual fit for special education settings (e.g., Simonsen et al., 2010) were also consulted. The use of this evidence‐informed a priori defined multicomponent framework, when coding the PBS components and characteristics in studies, allowed for an over‐estimation of their presence to be prevented. Approaches used were defined as PBS if: (i) it was clearly stated in the study that the intervention(s) in question had been implemented as part of a PBS framework used within that setting, and/or (ii) if they exhibited sufficient components that aligned with the PBS framework (Table 1). To be defined as PBS, interventions and supports had to include at least the primary characteristic of each PBS component. Therefore, the included studies had to report on the implementation of a proactive constructional approach utilised to establish consistent supportive contexts and environment. The approach also had to exhibit understanding of the function(s) of behaviour(s) to inform behavioural support procedures (including skills teaching), and thus design functionally informed supports. In addition to the primary characteristic of each PBS component, interventions and supports could also have any number of the remaining characteristics (secondary characteristics).
3. The types of studies that were eligible for inclusion included experimental designs (single case experimental designs or group designs), non‐experimental designs with a control group, and single group studies with baseline data without a control group. Any study reporting on quantitative data or qualitative data of relevance to the description and effectiveness of the PBS framework in special education settings delivered to CYP with developmental disabilities, the facilitators and barriers to the PBS framework implementation, and the views of stakeholders about PBS, was also eligible for inclusion.
4. Reported outcome was change in behaviours that challenge from baseline to the last available follow‐up point with any validated scale or defined observational approach (or perceived changes in behaviours that challenge for qualitative research)‐ using Emerson's (2001) definition:
  Culturally abnormal behaviour(s) of such an intensity, frequency or duration that the physical safety of the person or others is likely to be placed in serious jeopardy, or behaviour which is likely to seriously limit use of, or result in the person being denied access to, ordinary community facilities. (p. 3)

Additional outcomes reported could pertain to: (i) quality of life, mental health, or any other outcome for CYP with developmental disabilities; (ii) dimensions of PBS used in the interventions (e.g., characteristics of the PBS framework that underlie the interventions); (iii) stakeholder perspectives (e.g., experiences and views) on PBS implementation; or (iv) perceived facilitators and barriers to implementation of the PBS framework. Facilitators to PBS implementation were defined as a ‘practice, policy, or characteristic of the organization that functioned to increase or improve adoption of the Positive Behavioral Interventions and Supports framework’, whereas barriers to PBS implementation were defined as a ‘practice, policy, or characteristic of the organization or personnel that hindered implementation of the Positive Behavioral Interventions and Supports framework’ (Swain‐Bradway et al., 2013, p. 36).

**TABLE 1:** Positive behavioural support multicomponent framework

Components	Primary characteristics	Primary (in bold) and secondary characteristics
Values	Proactive constructional approach	**V1. Proactive constructional approach**, focusing on building social and other functional competencies (skills‐building) or increasing life opportunities. (Skills‐building and increased life opportunities to improve Quality of Life are a focus in their own right and are the primary outcome/goal of PBS implementation. An additional effect of this focus is that the likelihood of behaviour that challenges will be reduced, which is effectively then a secondary outcome/goal of PBS implementation.)
		V2. Socially valid person‐centred supports that are respectful, non‐aversive and focus on the Quality of Life (QoL) and wellbeing of service users as the primary outcome.
		V3. Stakeholder participation/involvement at every step of the PBS implementation.
Systems	Supportive contexts and environment	**S1**. Application of a continuum of support at the individual level as a minimum, to establish consistent **supportive contexts and environment** across various settings, providers of support (including family carers), and time. (Systems of Support‐ Individual Level Supports)
		S2. A systems approach that establishes ecologically valid supports that are a good contextual fit at the individual, group(s), or whole setting level (ideally across all levels) via appropriate organisational infrastructure. (Organisational Systems‐ Systems Redesign)
Science and Technologies	Understanding the function(s) of behaviour(s) and designing functionally informed supports	**S&T1.** Functional Assessment to facilitate an **understanding of the function(s) of behaviour(s) and inform behavioural support procedures** (including skills teaching). (Function of Behaviour)
		S&T2. Evidence‐informed practices for assessment and intervention, deriving primarily from behaviour analysis. (Practices)
		S&T3. Data‐driven processes of decision‐making and problem‐solving for intervention design, monitoring, and evaluation. (Data)
		S&T4. Use of complementary evidence‐based approaches may be incorporated to address key causal factors. (Complementary Approaches)

Abbreviation: PBS, positive behavioural support.

e) Any comparison intervention including: (i) support‐as‐usual control group; (ii) waiting‐list control group; (iii) active control group; or (iv) any other intervention control group; or (v) change from baseline data (pre‐intervention measures) compared to post‐intervention measures with no control group or condition.

f) PBS in special education settings (special school or college, special education classroom or unit in a mainstream school or college) where classes take place during the school day.

g) No restrictions were applied for the demographic characteristics (other than age and diagnosis) of the participants.

h) Only articles published in the English language were included in the systematic review. However, no restrictions were applied considering the country where the studies were conducted. There was also no restriction regarding publication date, thus the date range was not decided a priori.

### 2.3 Exclusion criteria

Studies were not included if they did not meet the inclusion criteria, or if they were reviews, or opinion articles, or studies not reporting any quantitative or qualitative data, or studies not reporting on sufficient methodological information (such as studies that did not meet the required standards of demonstrating intervention effectiveness across at minimum three different data points: multiple baseline design across only two participants, settings, or behaviours, and AB case studies with only one participant, etc.).

### 2.4 Review strategy

**FIGURE 1:**
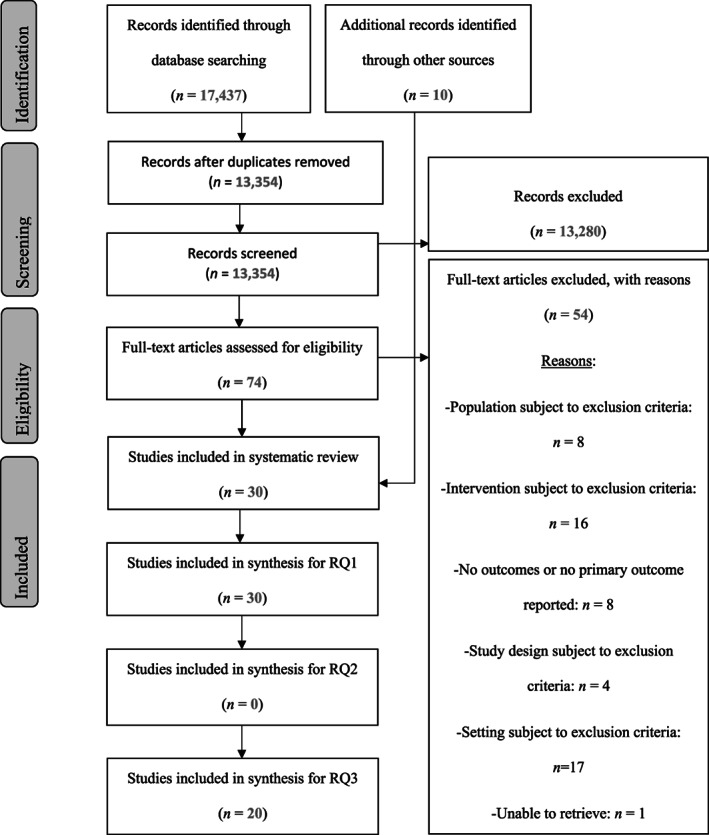
Preferred reporting items for systematic reviews and meta‐analyses flow diagram. Thirty studies were eligible for addressing review question 1 (RQ1), of which 20 were also utilised to address review question 3 (RQ3), and zero studies were eligible for addressing review question 2 (RQ2)

Electronic searches were conducted (see Figure 1: PRISMA Flow Diagram) and the identified records were exported to the EndNote reference software, which was used for the management of references. After deduplication, title and abstract records were screened by the first author. The fourth author independently screened 1500 randomly selected records, verifying inclusion/exclusion for full text screening, which resulted in 1490 agreements (inter‐rater reliability 99.33%, Cohen's *k* = 0.811). Following the title and abstract (stage 1) screening, full‐text screening was conducted. The fourth author verified inclusion/exclusion eligibility for full texts following a two‐step screening process. First, 15 records were screened resulting in 12 agreements (interrater reliability 80%, Cohen's *k* = 0.526). Following revision of the inclusion criteria guide used for full text screening, 10 additional records were screened by the second reviewer, which resulted in 9 agreements (interrater reliability 90%, Cohen's *k* = 0.800). Disagreements were discussed and resolved.

### 2.5 Data extraction

For the data extraction, a bespoke Excel form was used to record the information collected from eligible studies. The data included information about the study (authors, year, and country where the study was conducted); information about the number and demographic characteristics of CYP (gender, age, ethnicity/race, socioeconomic status, grade/school year, diagnosis, IQ or level of ability, skill deficits, and behaviours that challenge); information about stakeholders (age, gender, socioeconomic status, prior experience, job role, and relation to the CYP people population); data relating to the special education setting (e.g., special school, resource room, specialist provision classroom, other type); and support providers: CYP ratio.

For RQ1, data were extracted on the interventions implemented, the components of the PBS framework reported for the intervention, and the outcomes. Extracted data included PBS framework components and characteristics (Table 1), intervention characteristics (types, focus, tier of support, duration, number and length of sessions, intervention level, intervention provider, fidelity data), and training as well as supervision, that were related to PBS implementation. Extracted outcomes included: (a) primary outcomes: changes in behaviour that challenges, and (b) secondary outcomes: quality of life, adaptive skills, academic achievement, mental health, and other secondary outcomes. For RQ2, data were to be extracted on reported facilitators and barriers, as well as data collection methods for these factors. For RQ3, data were extracted on experiences of stakeholders, level of social validity data, data collection methods, and respondents. Data required for the quality appraisal were also extracted.

### 2.6 Data synthesis

Both quantitative and qualitative data available were narratively synthesised. For RQ1, a narrative synthesis was conducted focusing on the description of the PBS components implemented in special education settings, and the effectiveness of interventions. A narrative synthesis was also undertaken for RQs 2 and 3, focusing on facilitators and barriers of PBS implementation and the experiences of stakeholders with PBS implementation, respectively.

For conducting the quality appraisal of the identified studies, appropriate Critical Appraisal Skills Programme (CASP, 2018) checklists or the Single Case Study Risk of Bias tool (SCD RoB; Reichow et al., 2018) for single subject research designs were used. The latter critical appraisal tool (SCD RoB) is based on the Cochrane classification of bias: (a) selection bias; (b) performance bias; (c) detection bias, and d) ‘other’ types of bias (Reichow et al., 2018). Selection bias refers to the systematic differences between the characteristics of the participants during baseline conditions such as the allocation procedures and the selection criteria of the participants. Performance bias refers to systematic differences between the participants in areas related to the intervention and factors other than the intervention, such as blinding of personnel and participants, and procedural fidelity. Detection bias refers to systematic differences between participants in procedures for determining outcomes such as blinding of outcome assessors, outcome reporting and reliability, and data sampling. ‘Other’ types of bias refer to additional sources of bias relating to the area of research that cannot be classified under the previous categories of bias. The choice of a suitable methodological tool selected to appraise the quality of the studies was dependent on the research design of each study. The CASP critical appraisal checklist for randomised control trials without the randomisation questions was used for the group study included, and the SCD RoB was used for the single case studies.

The quality indicators used by Spear et al. (2013) reporting on social validity, as defined by Horner et al. (2005), were utilised for the evaluation of the social validity of the studies. The quality indicators include four main quality indicators and additional sources of social validity. The first quality indicator is related to social importance of the intervention goals and desirability of the goals by society. The second quality indicator is related to social importance of the change in the dependent variable as a result of the intervention implemented. The third quality indicator is related to the feasibility and cost‐effectiveness of the intervention. The fourth quality indicator is related to the long‐term implementation of the intervention by typical providers of supports using available resources in typical contexts. Additional measures of social validity included generalisation, maintenance, and explicit measurements of the intervention's social validity.

## 3 RESULTS

### 3.1 Study selection

The electronic searches identified 17,437 records (see Figure 1: PRISMA Flow Diagram). After deduplication, 13,354 title and abstract records were screened by the first author. The fourth author independently screened 1500 randomly selected records, verifying inclusion/exclusion for full text screening. Following the title and abstract (stage 1) screening, a total of 74 articles were selected from the database searches for full‐text screening, and from these, 54 articles were excluded (see Figure 1 for exclusion reasons). The fourth author verified inclusion/exclusion eligibility for full texts. A total of 20 articles, identified from the database searches were included in the data extraction phase. Ten additional articles were identified after the backward and forward searches, and the hand search of the IJPBS. Thirty articles were thus included in the systematic review, of which all of them were utilised to answer RQ1, 20 were utilised to answer RQ3, whereas no papers were eligible for answering RQ2. The characteristics of the included studies are summarised in Tables 2 and 3, and more details are provided in Tables S2 and S3.

**TABLE 2:** Summary of the PBS framework characteristics in interventions in studies addressing RQ1

	Number of studies (percentage %)	
PBS framework characteristics	Reported	Not reported
*Values*		
V1	30 (100%)	0 (0%)
V2	30 (100%)	0 (0%)
V3	30 (100%)	0 (0%)
*Systems*		
S1	30 (100%)	0 (0%)
S2	20 (66.67%)	10 (33.33%)
*Science and Technologies*		
S&T1	30 (100%)	0 (0%)
S&T2	30 (100%)	0 (0%)
S&T3	30 (100%)	0 (0%)
S&T4	17 (56.67%)	13 (43.33%)

Abbreviation: PBS, positive behavioural support.

**TABLE 3:** Characteristics of studies addressing RQ3

Authors (year; country)	Methods	Findings
Artman‐Meeker et al. (2017) (USA)	Questionnaire (6 item satisfaction survey) and 4 open‐ended questions completed by school pre‐service behaviour analysts	Participants reported Bug‐In‐Ear coaching improved use of Functional Communication Training and would recommend it. Coaching was somewhat distracting, but less disruptive than other coaching. Two participants had difficulties working with multiple students.
Banda et al. (2009) (USA)	Questionnaire and qualitative responses by TA and mother	Mother was pleased and reported that child enjoyed work and was less resistant to work with service providers. TA reported child was calmer.
Banda et al. (2012) (USA)	Teacher, TA, parent completed intervention Rating Profile‐15 (Martens & Witt, 1982). TA and parent gave qualitative responses	Teacher strongly agreed with 11 items (*M =* 5.67; range 4–6), TA with 12 items (*M* = 5.73; range 4–6), and parent with 13 items (*M* = 5.87; range 5–6). TA reported that student occasionally held blanket but relinquished without self‐injury, and parent was pleased with progress.
Bethune and Wood (2013) (USA)	Likert scale questionnaire and open‐ended section completed by teachers, and modified questionnaire for school psychologist	Intervention: Four teachers strongly agreed for importance and continued use, psychologist strongly agreed for importance. Three teachers and psychologist strongly agreed, and one teacher scored 3 for effectiveness. Coaching: Three teachers strongly agreed it is non‐intrusive, acceptable, effective, cost‐efficient, and psychologist too except for 1st one (rated 4).
Butler and Luiselli (2007) (USA)	Anecdotal data in discussion by researcher on staff experiences.	Intervention was feasible (easily integrated into classroom activities) and acceptable (well‐received) by staff at the educational setting.
Calloway and Simpson (1998) (USA)	Anecdotal data for socially important goals of intervention.	The social importance of the goals chosen for each student and rationale behind the choice of the target behaviours were reported informally.
Cavalari et al. (2014) (USA)	Anecdotal researcher‐reported data in discussion for staff experiences with intervention.	Infrequent skin picking in follow‐up, thus durable results. Stakeholder participation, staff expertise and student's school attendance aided intervention consistency and fidelity but no generalisation at home.
Cihak and Gama (2008) (USA)	Intervention Rating Profile‐15 (16 items) teacher‐completed.	Procedures suitable and liked by teachers, intervention was fair, they would suggest it and it had no negative side‐effects for the child.
Clarke and Duda (2019) (USA)	Five‐point scale completed by peer buddies pre‐ and post‐ intervention on modified quality of life indicators for student, and direct observation data collected on ‘positive affect’ of student.	Increased ‘positive affect’ from *M* = 8% (range 0–27%) in baseline and withdrawal sessions to *M* = 26% (range 4–33%) during intervention. Mean peer buddies' social validation ratings were higher after intervention (‘Friendships with her peers now are’: 4–4.6; ‘Mia's relationships with her teachers’: 3.3–4.2; ‘general happiness’: 3.3–3.6; for ‘behavior is appropriate’: 2.8–3.4).
Dunlap et al. (1995) (USA)	Six‐point Likert scales (Dunlap, 1984) to assess from videotapes interest and happiness of student.	The intervention targeted meaningful outcomes (reduced challenging behaviours, increased on‐task behaviour, increased happiness, and interest of student)
Flynn and Lo (2016) (USA)	Teachers completed adapted version of Teacher Post‐Intervention Acceptability and Importance of Effects Survey (Lane & Beebe‐Frankenberger, 2004; 11 items), Likert for trial‐based Functional Analysis (TBFA) and intervention, two open‐ended questions on what they like and what needs change	Teachers agreed or strongly agreed that TBFA and intervention were easy to learn (*M* = 4.0) and perform (*M* = 4.3), would conduct TBFA (*M* = 4.7) and intervention with other students (*M* = 4.3), and would recommend TBFA (*M* = 4.0) and intervention (*M* = 4.7). Two teachers agreed or strongly agreed that intervention increased students' replacement behaviours (*M* = 3.7) and reduced challenging behaviours (*M* = 3.7), while one teacher neither agreed nor disagreed (‘3’). Open‐ended questions: Training and feedback were beneficial (Teachers 1 and 3), ‘understanding behavioral function was useful’ (Teacher 2), implementation of intervention produced positive outcomes.
Friedman and Luiselli (2008) (USA)	Non‐systematic social validity data obtained by staff reports.	Staff reported that intervention was acceptable, and they were pleased with quick response of student. Authors suggested staff satisfaction could explain continued use.
Lalli et al. (1993) (USA)	Researchers mentioned social validity data in discussion section.	Teachers taught students alternatives, targeting socially important goals. Intervention had meaningful outcome (frequent interactions).
Lane et al. (2006) (USA)	Teacher and assistant completed Intervention Rating Profile‐15 (IRP‐15; Martens et al., 1985), and student Child Intervention Rating Profile (CIRP; Witt & Elliott, 1985).	IRP‐15 social validity scores ranged from 15 to 90, and CIRP scores ranged from 7 to 42, with higher scores indicating higher treatment acceptability. Teacher and assistant rated intervention favourably (IRP‐15: 74 and 80, respectively), with slightly increased ratings after intervention (75 and 86, respectively). Student rated intervention favourably (CIRP: 38 at both times).
Larkin et al. (2016) (USA)	Treatment Acceptability Rating Form completed by teachers for intervention and assessment (Langthorne & McGill, 2011)	Teacher responses on the Treatment Acceptability Rating Form showed high acceptability for assessment and intervention. Teachers noted willingness to use the procedures again. Teachers also noted that the procedures had a positive impact on the behaviour of students.
Moore et al. (2009) (USA)	Anecdotal staff reports mentioned by researchers (no formal data)	Anecdotal reports on intervention and involvement were positive. Interventions were acceptable for school, useful and practical without researcher.
Mueller and Kafka (2006) (USA)	Teacher reported data (non‐systematic social validity data)	Teacher reports reflected positive perceptions about the effectiveness of the intervention and its ease of use, as well as its time requirements.
Mueller and Nkosi (2007) (USA)	IRP‐15 completed by teacher and paraprofessional after training	IRP‐15 results were 73 for teacher and 78 for paraprofessional. Results demonstrate that multicomponent intervention was highly acceptable.
Pennington et al. (2012) (USA)	Informal teacher reported data	Teacher reported that procedures were easy to implement and positive feelings about the level of participation of the student increased.
Pitts et al. (2019) (UK)	Questionnaire (5‐point scale) on the social validity of intervention and training completed by 14 school staff (teachers and teaching assistants)	Staff reported sufficient training (*M* = 4.5), focus on increasing positive behaviours (*M* = 4.5), meaningful goals (*M* = 4.5), feeling comfortable implementing 1:1 sessions (*M* = 4.5), and most disagreed that ABA induced pressure (*M* = 2.6). All agreed there were benefits for students (*M* = 5), assessments were appropriate, and lessons sufficiently planned (*M* = 4.85), there was regular monitoring (*M* = 4.78) and targets were adjusted (*M* = 4.64).

### 3.2 Quality appraisal

The risk of bias for participant selection and selective outcome reporting was low, while for procedural fidelity it was high in 50% (*n* = 15) of the studies. The blinding of participant and personnel was ‘unsure’ in all studies, and the blinding of outcome assessors was either unsure or high. When considered individually, no single‐case design study had an overall high risk of bias. Even when a study showed high risk for a specific type of bias, it still had either low or unsure risk for most other types of bias. The literature on PBS implementation in special education settings appears, therefore, to include single‐case studies of generally acceptable quality.

The one group study included in the review that used a pre and post intervention within‐group design (Pitts et al., 2019), addressed a focused issue, the participants were all accounted for in the conclusion, and the application of the results to the local population or in a particular context (e.g., special education setting) was possible. Clinically important outcomes were addressed but only partly, as important outcomes such as mental health and quality of life were not measured. It was uncertain if blinding was applied because there was no report about its absence or presence. The intervention had significant positive effects for certain behaviours that challenge, such as self‐injury and stereotypy, and non‐statistically significant effects for other types of behaviours, such as aggression. More details on the methodological quality and the quality indicators can be found in the Tables S4 and S5.

### 3.3 RQ1: Implementation of the PBS framework in special education

The studies included reported on interventions to support CYP with an intellectual disability or genetic conditions generally manifesting with intellectual disability (15%), or with autism (34%), or having multiple diagnoses including autism and/or intellectual disability (51%). Publication dates of the studies were between 1993 and 2019. Most were conducted in the USA (*n* = 27, 90%) with three studies conducted in the UK (*n* = 3, 10%).

Five studies (16.67%) were multiple baseline design studies, 19 studies (63.33%) used reversal designs, and one study was labelled as a quasi‐longitudinal (quasi‐experimental) descriptive case study that had an ABA design (Moore et al., 2009). Moreover, one study included a case series approach (Foran et al., 2015), one utilised an alternating treatments design (Lang et al., 2010), and one study utilised both reversal and multiple baseline design elements depending on the setting in which the intervention was implemented (Mueller & Nkosi, 2007). One study was described as using single‐subject design with multicomponent interventions for three children (Paris et al., 2019), and one study included a single group study design with pre and post intervention measures (Pitts et al., 2019). Details of the PBS characteristics of the reported interventions can be found in Table 2.

### 3.4 Description of interventions following a PBS framework

The majority of the interventions implemented included a combination of antecedent‐based and consequence‐based strategies (*n* = 21, 70%), with almost all the remaining interventions including antecedent‐based strategies (*n* = 7, 23.33%), except for two that included only consequence‐based strategies. The range of interventions included Functional Communication Training/Teaching (FCT), Non‐Contingent Reinforcement (NCR), Differential Reinforcement, choice provided, environmental adjustments, and so on. Complementary interventions, such as social stories, visual communication tools, gestures and signing, and drug therapy were also used. All the interventions exhibited the features of Tier 3 specialist supports but only one study (Paris et al., 2019) explicitly labelled the tier of support implemented (Tier 3 for all participants). Interventions reporting sufficient information about the level of implementation were at an individual level, although in 11 studies (36.67%) interventions were delivered in the context of group instruction targeting only the individual participant in need for support. Detailed information on the interventions can be found in Table S2.

#### 3.4.1 Primary characteristics of PBS

Each included study had been selected for the presence of the three primary characteristics belonging to the Values, Systems, and Science and Technologies (Table 1) components. In total three of the studies (10%) exhibited seven PBS characteristics, 17 (56.67%) exhibited eight characteristics, and 10 studies (33.34%) exhibited all nine PBS characteristics. However, even the studies that included all nine PBS characteristics did not always report all elements of each characteristic. For example, the Systems primary characteristic was coded present if consistency was reported across either time, or support providers, or settings, or two or more of these elements. Consistency was not always reported across all three elements of time, support providers, and settings.

#### 3.4.2 Secondary characteristics of PBS

##### Values

The presence of the two secondary characteristics belonging to the Values component were identified in all the included studies, although not all the individual elements were reported. More specifically, the second characteristic of the Values component related to social validity of the approach was reported, and in some cases formally measured, in 20 studies (66.67%; which were included and analysed further for RQ3; Table 3). The third characteristic of the Values component related to stakeholder participation was also present in all studies. The commonly involved stakeholder group was school staff. If parents were involved it was mainly during FBA procedures, and if students were involved it was usually for input on reinforcers during preference assessments. All individual elements of the secondary characteristics of the Values component involving (a) assessment of the quality of life that was increased by the establishment of socially valid person‐centred supports, which were non‐aversive and respectful, and (b) stakeholder participation, were reported in two studies (Clarke & Duda, 2019; Dunlap et al., 1995).

##### Systems

In total, 20 studies (66.67%) reported on appropriate organisational infrastructure, as part of a systems approach, to establish ecologically valid supports that exhibit contextual fit. Appropriate organisational infrastructure within the educational setting refers to the supports in place (e.g., training, coaching, performance assessments, supervision, team meetings, and materials such as adherence checklists) for assisting support providers to offer high quality of services. To achieve the required systems change, appropriate organisational infrastructure should be in place, which will increase capacity for and support of the change. These types of supports are related to suitable protocols and policies of the organisation/school that operationalise its vision, effective and efficient processes, resources, skills and roles of staff members, and the external supports for systems change to scale‐up implementation capacity. These promote buy‐in of support providers and scale‐up of the approach to a school‐wide level.

##### Science and technologies

The second and third Science and Technologies secondary PBS characteristics, related to function‐informed behaviour analytic interventions and data‐driven processes, respectively, were reported across all studies (100%). The fourth characteristic, related to complementary evidence‐based approaches, was reported in 17 (56.67%) of the studies included.

#### 3.4.3 PBS implementation processes

Two of the included studies (Clarke & Duda, 2019; Paris et al., 2019) explicitly identified the implemented approach as PBS by naming it as PBS, and described the procedures involved during the implementation of a PBS framework in two special education settings. Clarke and Duda (2019) described the presence of a PBS team and facilitator, conducting FBA, hypotheses development, generation of a behaviour support plan (BSP), and the collection of progress and outcome data utilised for monitoring the intervention. Training offered to providers of supports prior to implementing the intervention was also evident and reported clearly in the study. In the Paris et al. (2019) study the steps illustrated included conducting an FBA, which led to hypotheses development, generating function‐based BSPs, conducting staff training, implementing the intervention, and conducting evaluation procedures by collecting progress and outcome data. Additionally, although not included in the illustrated steps of the PBS implementation procedures, a team‐based approach including teachers and paraprofessionals was highlighted in the text of the article (Paris et al., 2019). Overall, the two studies outlined a similar set of specific steps delivering a PBS framework in the context of special education settings.

#### 3.4.4 Effectiveness of PBS interventions in special education settings

Twenty‐eight of the 30 included studies (93.33%) reported considerable reductions in behaviours that challenge, without any increases being evident in any specific types of behaviours that challenge; they also reported increases in alternative behaviours taught if they were part of the intervention. It was not possible to include percentages of reduction in challenging behaviours for all studies, as not all studies provided sufficient data for this to be calculated. Overall, 26 studies (86.67%) demonstrated improvements in all measured outcomes in the form of reductions in behaviours that challenge, and additionally increases in alternative behaviours or adaptive skills, if these were part of the intervention outcomes measured. Four studies (13.33%) showed a mixed pattern regarding outcome improvements with either improvements in alternative behaviours but also increase in behaviours that challenge (Artman‐Meeker et al., 2017); or increase in certain behaviours that challenge and decrease in others paired with increase in adaptive skills for some participants while others remained stable (Paris et al., 2019); or statistically significant decrease in certain behaviours that challenge and non‐statistically significant decrease in others (Pitts et al., 2019); or decrease in behaviours that challenge and improvement in some alternative behaviours and not on other alternative behaviours for some participants (Bethune & Wood, 2013).

For the studies reporting percentages of reduction, four studies showed up to 20% decrease in behaviours that challenge, seven studies exhibited more than 20% decrease, and seven studies showed about 50% or more decrease. The group study (Pitts et al., 2019) showed statistically significant results for decrease in stereotypy with large effect sizes and in self‐injurious behaviours with medium effect sizes. For adaptive behaviours, there was up to a 20% increase in four studies, in four studies there was more than 20% increase, and in four studies there was a 50% or more increase. Two studies (Artman‐Meeker et al., 2017; Paris et al., 2019) demonstrated mixed results for the effectiveness of the interventions. Paris et al.'s (2019) Tier 3 case studies in a special school for students with severe intellectual disabilities showed reductions for certain behaviours, such as aggressive behaviours, but no difference or a slight increase in other behaviours, such as stereotypy. In contrast to the majority of studies, Artman‐Meeker et al. (2017) reported increase in behaviours that challenge for two students, while the third student exhibited almost no behaviour that challenges during the study, but coaching was associated with increases in their communication.

From the 30 studies, 17 (56.67%) reported the measurement of secondary outcomes in the form of mostly replacement behaviours, but also communication skills, quality of life indicators, and learning and academic skills. The two studies explicitly identifying the approach implemented as PBS (while the remaining studies did not explicitly name the approach as PBS even though it aligned with the PBS framework) measured quality of life indicators as reported by peers, positive affect, and engagement of the student supported, demonstrating increased levels of these secondary outcomes (Clarke & Duda, 2019), and also measured communication, daily living skills, socialisation, and motor skills using the Vineland Adaptive Behaviour Scales (Paris et al., 2019), showing increased communication for two of the students participating. Quality of life was only assessed formally in two studies (Clarke & Duda, 2019; Dunlap et al., 1995), and informally in four more studies (13.33%) (Banda et al., 2009; Flynn & Lo, 2016; Foran et al., 2015; Hansen & Wadsworth, 2015) by taking into consideration anecdotal data. Quality of life was formally measured by assessing the happiness and interest of the student as rated by researchers after watching videotaped sessions (Dunlap et al., 1995), and by assessing positive affect of the student after observation of positive affect behaviours and after using quality of life indicators scores provided by peer buddies (Clarke & Duda, 2019). Engagement of students, including on task behaviour, measured in four studies (13.33%; Cihak & Gama, 2008; Clarke & Duda, 2019; Larkin et al., 2016; Pennington et al., 2012) and two studies (Bethune & Wood, 2013; Dunlap et al., 1995), respectively, was the measure most closely related to quality of life outcomes when an explicit measure of quality of life was not included.

### 3.5 RQ2: Perceived barriers and facilitators to the PBS framework implementation

No studies reported data on facilitators and barriers for the implementation of a PBS framework in special education settings to support CYP with developmental disabilities.

### 3.6 RQ3: Experience of PBS framework implementation

For answering RQ3, 20 studies (66.67%) out of the 30 studies included in the systematic review were utilised (Table 3) and 12 of these 20 studies (60%) reported formally collected social validity data, either quantitatively (for seven of the eligible studies; 35%), or both quantitatively and qualitatively (for five of the studies; 16.67%) collected, with the remaining studies including anecdotal data only. Quantitative data were collected by utilising questionnaires and surveys, sometimes supplemented with open‐ended questions (in three studies; 15%) or qualitative responses (in two studies; 10%). No qualitative‐only studies investigating the experiences of stakeholders of PBS to support CYP with developmental disabilities were identified. Social validity data respondents were usually teaching staff (for 14 of the eligible studies; 70%), with two studies (10%) collecting parental data on social validity (Banda et al., 2009; Banda et al., 2012), one study collecting peer perceptions on quality of life indicators (Clarke & Duda, 2019), two studies (10%) utilising observational data to assess the positive affect of CYP with developmental disabilities (Clarke & Duda, 2019; Dunlap et al., 1995), and only one study (Lane et al., 2006) collecting student provided social validity data.

School staff views were in general favourable towards the interventions. Parental, peer, and student views indicated that they also perceived the interventions implemented as socially valid. Perceptions of stakeholders on training and coaching were investigated in four studies (20%; Artman‐Meeker et al., 2017; Bethune & Wood, 2013; Flynn & Lo, 2016; Pitts et al., 2019), and were generally positive. Broader dimensions of the experience of stakeholders with PBS interventions were not reported.

## 4 DISCUSSION

The main aim of the current systematic review was to review, synthesise and critically appraise the available evidence on PBS implementation within special education settings to support CYP with developmental disabilities. A meta‐analysis was not part of this systematic review. This is because, as outlined in the protocol, the most significant question that the review aimed to address was the description of the components of PBS that have been used in interventions in special education settings. A narrative synthesis was therefore more appropriate. Moreover, the review aimed to address the description of the facilitators and barriers to PBS implementation, and social validity/experience of the interventions, which also required a narrative synthesis. In addition, the measurement of outcomes exhibited heterogeneity across the studies, preventing a meta‐analytic synthesis.

The findings on the outcomes of PBS interventions suggest that they are generally effective in increasing adaptive behaviours and decreasing behaviours that challenge of CYP with developmental disabilities in special education settings. The lack of more robust high quality methodological designs employing a control group, such as randomised controlled trials, and the heterogeneity of outcome data across the included studies, make it difficult to assess precisely the effectiveness of PBS in special education settings. The majority of the included studies that employed a single‐case study design were generally of acceptable quality. However, there were elements of single‐case study designs, such as blinding procedures and procedural fidelity, for which the risk of bias was on average unsure or high. These types of bias in studies of PBS implementation in special education settings contribute directly to reduced certainty in the available effectiveness evidence.

It is worth noting that indicators of quality of life, an outcome that PBS aims to promote, were only assessed formally in two studies and informally taken into consideration in four more studies. Considering the emphasis that the PBS framework places on quality‐of‐life outcomes, the lack of evidence of reporting of these outcomes is disappointing. All the more so given that in one of the first attempts to systematically synthesise the literature base on PBS more than 20 years ago, Carr et al. (1999) emphasised the need for research to include measures of comprehensive lifestyle change and not only reductions in behaviours that challenge. More than 20 years on, the need for including standardised measures related to quality of life to assess outcomes of PBS interventions remains. While the limited number of standardised methodological tools measuring quality of life may contribute to this, the results of this review show that it is possible. Overall, additional outcomes reported were present in only 17 studies (56.67%), suggesting the need for assessing and reporting multiple outcomes (lifestyle changes, skill development, etc.) when implementing PBS, and not just behaviour that challenges.

All nine characteristics of PBS (as defined in the framework used in this review) were present across only 10 studies (33.33%), as not all studies included reporting of all secondary characteristics. The two secondary characteristics of the Values component, namely non‐aversive, respectful and socially valid person‐centred supports, and stakeholder participation, and the secondary characteristics of the Science and Technologies component related to evidence‐based practices deriving primarily from behaviour analysis and data‐driven processes were reported in all 30 studies (100%). One of the two secondary characteristics most often neglected was the reporting of a systems approach to establish valid supports that are a good contextual fit at the individual, group(s), or whole setting level (ideally across all levels) via appropriate organisational infrastructure. The other secondary characteristic (in the Science and Technologies component) was the use of complementary evidence‐based approaches. These were reported in 20 studies (66.67%) and 17 studies (56.67%), respectively. This suggests that interventions either lacked or researchers did not report important PBS features (which may suggest a need for reporting standards in PBS intervention research). Absence of PBS characteristics, which are necessary to ensure high quality support, can decrease the structural integrity of the approach.

Two studies, one with low and one with unsure risk of bias, noted the procedures followed for PBS implementation with clear steps in chronological order. Therefore, there was some evidence that PBS can be successfully delivered in special education settings when team collaboration is in place. However, none of the included studies explicitly assessed facilitators and barriers to implementation. Therefore, RQ2 could not be addressed. An evidence base about implementation is essential when trying to ensure stakeholder buy‐in, consistent PBS delivery and sustainability, and take‐up of PBS in special education settings for CYP with developmental disabilities. Data on experiences with PBS implementation can be beneficial in improving support for practitioners, and hence aid successful PBS implementation.

Stakeholders' experiences suggested generally favourable views about PBS interventions: considering the goals meaningful, the procedures acceptable, and the outcomes important. Social validity data were typically gathered from staff and sometimes parents, but data from students must be included in future research. Most of the studies addressing RQ3 regarding the experiences of stakeholders were, on average, studies with unsure risk of bias. However, 75% of the 20 studies addressing RQ3 included the presence of more than half of the quality indicators reporting on social validity. Therefore, they did show acceptable levels of reporting information on social validity indicators. However, the detail of the evidence reported in these studies was limited; highlighting the need for more detailed high‐quality studies on experiences of stakeholders focused on PBS implementation. No comprehensive qualitative studies investigating experiences of stakeholders were identified, indicating a significant evidence gap. Just as PBS should involve stakeholders during implementation, it would be equally beneficial for stakeholders to be included in research on PBS that can impact practice, as they can provide valuable insights on PBS implementation and reveal any difficulties that need to be addressed.

This systematic review focused specifically on studies conducted in special education settings to support CYP with developmental disabilities that aligned with a PBS framework, exhibiting primary PBS components and sufficiently reported a function‐informed approach, which is at the core of PBS. However, there is a wider literature on PBS worth noting although not included, as it was subject to exclusion. Studies, for example, that utilised multiple baseline methodology but only across two conditions, were not eligible for inclusion due to not meeting methodological standards in line with recommendations of Horner et al. (2005). This also meant that most studies included in the review should have been more likely to already be of a generally acceptable level of quality. Studies that did not include sufficient methodological information such as information on baseline measures (Jackson Brown et al., 2014), or reported only on outcomes such as adaptive skills and behaviours (Lambert‐Lee et al., 2015; Wadsworth et al., 2015), were not eligible for inclusion.

Across all studies, the use of standardised tools to assess PBS implementation was not reported. Although methodological tools for assessing PBS implementation are available (OSEP Technical Assistance Center on Positive Behavioral Interventions and Supports, n.d.), these have been created for mainstream schools and likely require adaptations so that they can be appropriate for special education settings. Adaptations may need to reflect the unique adaptations of the PBS framework to support CYP with developmental disabilities in special education settings, so that contextual fit is ensured.

### 4.1 Limitations

In terms of limitations, these can be identified at the review level and at the level of individual studies included in the systematic review. At the review level, one article (Peterson et al., 2002) considered during the abstract screening stage as eligible for proceeding to full text screening could not be retrieved. Therefore, we cannot be certain if this study would have been included in the systematic review and impact on the current results or not. Moreover, the identification of studies describing the PBS framework was made possible by evaluating them against the components and characteristics of the framework, because not all studies explicitly named as PBS the approach they were following. This may have led to studies not being identified by the search strategy, especially in cases such as when reporting in the articles of the PBS framework primary components was inadequate.

At the study level, there were very limited group studies eligible for inclusion (one group study), and no randomised controlled trials. The small sample of this group study, the lack of a control group, and the absence of blinding or absence of reporting on blinding procedures decreased the quality of the evidence that the study provided for this systematic review. Moreover, the available data for addressing RQ3 were brief social validity data (provided by quantitative scales and sometimes supplemented with brief comments on open‐ended questions) most of which were associated with unsure risk of bias but acceptable level of reporting information on social validity related indicators, and no detailed qualitative studies were identified that could be utilised to address RQ2. Regarding the single‐case studies, there were certain types of bias in the risk of bias assessment, such as blinding of outcome assessors (that were consistently rated unsure for all studies), and procedural fidelity (which was high risk of bias in half of the studies included), that contributed to decreased methodological quality and thus certainty in the evidence from single‐case studies.

More group and qualitative studies are needed for PBS implementation in special education settings, which will describe additional to Tier 3 supports, that may be implemented in the setting, and will follow more robust designs. Additional consideration should also be given when designing and reporting single‐case studies regarding blinding procedures and procedural fidelity. These design and methodology considerations were most commonly vulnerable to risk of bias in the current review.

### 4.2 Implications and future directions

The findings of this systematic review suggest that PBS holds the potential for decreasing behaviours that challenge and increasing adaptive behaviours of CYP with developmental disabilities in special education settings. These findings extend the research base on PBS and are relevant for practitioners and administrators aiming to promote an increased quality of life for CYP with developmental disabilities, who can benefit from implementing PBS.

Although there is a wider literature on PBS in special and alternative settings, the evidence base on PBS implementation in special education settings to support specifically CYP with developmental disabilities remains insufficient for the target context and population. Future research should explore further the implementation of school‐wide PBS in special education settings by using high quality methodological designs, so that studies exhibit low risk of bias (especially for blinding procedures and procedural fidelity), and include more robust group designs, such as randomised controlled trials. Areas that future research should focus on are: (a) how PBS function‐based interventions are incorporated as part of a tiered model in special education settings to support CYP with developmental disabilities; (b) providing a comprehensive description of PBS framework characteristics, processes, and adjustments required to ensure contextual fit; (c) exploring facilitators and barriers to PBS implementation; and (d) employing more thorough qualitative measures to explore experiences of stakeholders with PBS and its social validity in such settings. Moreover, future research should include the assessment of quality‐of‐life changes, either as a central or as an additional dependent variable, when measuring the impact of PBS.

There are currently no official standards for reporting PBS interventions in research, and many of the studies included in the current review lacked information on whether certain characteristics of PBS were present. The generally poor reporting of details for psychological interventions (Premachandra & Lewis, 2021) necessitates the use of more consistent reporting standards. Using general reporting standards for psychological interventions (e.g., TIDieR; Hoffmann et al., 2014) in parallel with a framework for PBS implementation in special education settings, such the PBS framework in Table 1, for reporting features of PBS present in intervention studies can be useful until specific reporting standards for PBS studies are developed. PBS has a 40‐year long history of implementation, and yet a lack of clarity on how PBS is defined and reported in the evidence base, and issues related to its social validity remain present. These limitations should be addressed by future research.

Overall, the results of this systematic review suggest that PBS can be successfully implemented in special education settings to support CYP with developmental disabilities. Future research should explore further the implementation of PBS in a school‐wide level in special education settings, by employing high quality group designs and more comprehensive qualitative methods.

## Supporting information


**Appendix S1**: Supporting Information

## Data Availability

The data that supports the findings of this study are available in the supplementary material of this article.

## Footnotes

^1^
PBS refers to positive behavioural support

^2^
CYP refers to children and young people

^3^
FBA refers to functional behaviour assessment
