# Supplementary material for: Positive behavioural support for children and young people with developmental disabilities in special education settings: A systematic review
Source: J Appl Res Intellect Disabil. 2022 Feb 23;35(3):719–35. doi: 10.1111/jar.12989 (PMC9306923; doi:10.1111/jar.12989)
Supplement: Supplementary file 1 — Appendix S1: Supporting Information [file JAR-35-719-s001.docx]

**Supporting Information: Tables**

**Table S1*.*** *Example of search string*

| Search terms entered into the PsychINFO database |
| --- |
| 1. ((Learning or Intellectual* or Mental* or Neurodevelopmental* or Developmental* or "Childhood Disintegrative" or Genetic) adj3 (Disab* or Difficult* or Deficien* or Impair* or Disorder* or Handicap* or Delay* or Abnormal* or Subnormal* or Retard*)).ti,ab,mh,sh,hw. 2. Limit 1 to English 3. (ID or DD).ti,ab,mh,sh,hw. 4. Limit 3 to English 5. ("Profound* Intellectual* Multiple Disab*" or PIMD or "Profound* and Multiple Learning Disab*" or PMLD).ti,ab,mh,sh,hw. 6. Limit 5 to English 7. (Autis* or "Autis* Disorder" or "Autis* Spectrum Disorder*" or ASD or Asperger* or "Pervasive Developmental* Disorder*" or PDD or "Pervasive Developmental* Disorder* Not Otherwise Specified" or "PDD-NOS").ti,ab,mh,sh,hw. 8. Limit 7 to English 9. (("Smith-Magenis" or Rett* or "Lesch-Nyhan" or "Prader-Willi" or Angelman or "fragile X" or "Cri-du-chat" or "Cornelia de Lange" or "de Lange" or "Rubinstein-Taybi" or "Fetal alcohol" or velocardiofacial or Klinefelter or DiGeorge or Overgrowth or Asperger* or "22q11.2" or Shprintzen or Down*) adj3 syndrome).ti,ab,mh,sh,hw. 10. Limit 9 to English 11. ("Prenatal alcohol exposure" or "Static encephalopathy" or Neurofibromatosis or Hypothyroidism or Phenylketonuria).ti,ab,mh,sh,hw. 12. Limit 11 to English 13. ((Complex or "Special educat*" or Special* or "Educat* and behav*") adj3 (need* or "need* and disabilit*")).ti,ab,mh,sh,hw. 14. Limit 13 to English 15. (SEN or SEND).ti,ab,mh,sh,hw 16. Limit 15 to English 17. (abnormal* or subnormal* or retard*).ti,ab,mh,sh,hw. 18. Limit 17 to English 19. exp Intellectual development disorder/ 20. exp Learning disorder/ |
|  |

(*continued*)

**Table S1.** (*Continued)*

| Search terms entered into the PsychINFO database |
| --- |
| 1. 1 or 3 or 5 or 7 or 9 or 11 or 13 or 15 or 17 or 19 or 20 2. 2 or 4 or 6 or 8 or 10 or 12 or 14 or 16 or 18 or 19 or 20 3. ("Behav* of concern*" or "Behav* that challeng*" or Aggression or Violence or Self-injury or Self-harm or Disruption* or "Behav* difficult*" or Destruction* or "Student* discipline" or "Disciplin* problem*" or Tantrum* or "Behav* management" or "Behav* chang*" or "Social* difficult*" or Stereotyp*).ti,ab,mh,sh,hw. 4. Limit 23 to English 5. ((Challenging or Aggressive or Violent or Self-injurious or Problem* or Difficult* or Aberrant or Maladaptive or Inappropriate or Dangerous or Deviant or Disruptive or Antisocial or Undesirable or Destructive or Replacement* or Repetitive or Off-task or concern*) adj3 Behav*).ti,ab,mh,sh,hw. 6. Limit 25 to English 7. 23 or 25 8. 24 or 26 9. ((SEN or SEND or Special* or "Special education*") adj3 (school* or classroom* or class* or setting* or context* or provision* or unit*)).af. 10. Limit 29 to English 11. ((Alternative or Special* or self-contain*) adj3 ("educat* setting*" or setting* or school* or classroom* or class* or context* or provision* or unit*)).af. 12. Limit 31 to English 13. (Resource* adj3 (room* or classroom* or class* or base or context* or provision* or unit*)).af. 14. Limit 33 to English 15. exp Alternative school/ 16. 29 or 31 or 33 17. 30 or 32 or 34 18. 35 or 36 19. 35 or 37 20. 21 and 27 and 38 21. 22 and 28 and 39 |
|  |

| **Table S2**. *Detailed studies addressing RQ1* | | | | | |
| --- | --- | --- | --- | --- | --- |
| Authors (Year)  (Country) | Design & Outcome Measures | Participants (CYP) & Setting | Intervention | PBS Framework Components | Outcome |
| Artman-Meeker et al. (2017)  (USA) | Concurrent multiple baseline design across participants utilised to investigate:  1. Challenging behaviours rate (percentage intervals with CB)  2. Rate of communication | 3 males (4 years old, 4 years old and 5 years old) with autism (ASD) across 2 special education classrooms within a university-affiliated early childhood centre in the Pacific Northwest (ABA-based extended day program) | - Assessment of behaviour function: Motivation Assessment Scale (MAS; Durand, 1990), Functional Assessment Interview Form (FAI; O’Neill et al., 1997), direct observations (at least 20 min of semi-structured play time), Brief Functional Analysis: BFA (Northup et al., 1991) for assessing behaviour function (function: access to tangibles for all CYP) - Implementation of Functional Communication Training (FCT) to support children and young people utilising function-based communication | ***Values*:**  V1, V2, V3  ***Systems***:  S1, S2  ***Science & Technologies:***  S&T1, S&T2, S&T3  8 characteristics | 1. For Carter and Aaron CB rate increased in level and variability from *M*= 3.6% (range 0–6.59%) in baseline to *M*=5.29% (range 1.67–10%) of intervals in B-I-E Coaching Phase, and from *M*=5.43% (range 0–12.5%) in baseline to *M*= 15.31% (range 1.67– 29.25%) of intervals with CB during the B-I-E coaching, respectively. Zac displayed almost no challenging behaviours during baseline (*M*= 0%) or intervention (*M*= 0.2%; range 0– 0.94%)      1. Rate of communication increased from baseline to B-I-E Coaching phase for all students. |
| Banda et al. (2009)  (USA) | Reversal design (ABAC) utilised to investigate challenging behaviour (self-injurious behaviour: SIB) measured as percentage of SIB per minute frequency | 1 male (13 years old) with severe autism (ASD) and Tourette syndrome in a self-contained classroom separated by curtain from rest of special education classroom with students ranging in age from 13 to 22 | - Assessment of behaviour function: Motivation Assessment Scale (MAS; Durand & Crimmins, 1992) with the teaching assistant (TA), interview with mother, informal interviews with teacher and TA, Brief Functional Assessment Interview with TA, Functional Analysis (function: multiple- attention and access to tangibles, and possibly automatic) - Implementation of positive social attention every 10 seconds (may have served as NCR) and extinction multicomponent intervention | ***Values:***  V1, V2, V3  ***Systems:***  S1, S2  ***Science & Technologies:***  S&T1, S&T2, S&T3  8 characteristics | SIB reduced from 5.7 hits per minute (range 3.8-7.5) in baseline to 3.5 hits per minute (range 0-7) in phase C reaching 0 hits per minute at the end of the intervention. |
| Banda et al. (2012)  (USA) | ABAB reversal design utilised to investigate challenging behaviour (self-injurious behaviour: SIB) measured as the number of hits in 5-min sessions (frequency counts) | 1 male (14 years old) with severe autism (ASD) and Tourette syndrome in a self-contained classroom with students ranging in age from 13 to 22 | - Assessment of behaviour function: Functional Assessment Interview with mother and teaching assistant, Functional Analysis (function: automatic or multiple) - Implementation of access to self-restraint during tasks, fixed interval reinforcement schedule, and extinction multicomponent intervention | ***Values:***  V1, V2, V3  ***Systems:***  S1, S2  ***Science & Technologies:***  S&T1, S&T2, S&T3  8 characteristics | SIB reduced from 21.3 hits per 5-minute session (range 12–32) in baseline to 1.1 hits per 5-minute session (range 0–6) in the intervention fading phase, reaching 0 hits per session at the maintenance phase of the intervention (after 5 and 6 months) |
|  |  |  |  |  | *(Continued)* |
| **Table S2*.*** *(Continued)* | | | | | |
| Authors (Year)(Country) | Design & Outcome Measures | Participants (CYP) & Setting | Intervention | PBS Framework Components | Outcome |
| Bethune & Wood (2013)  (USA) | Delayed multiple baseline across participants for the 4 teachers and multiple baseline across participants for the 4 students to investigate:  1. Challenging behaviours rate (frequency of CB and percentage intervals with CB)  2.Replacement behaviour (percentage intervals)  3.Functional communication (occurrence) | 1 female (4 years old; trisomy 9 mosaicism), 1 female (8 years old; Down’s Syndrome), 2 males (5 and 10 years old; autism) in exceptional children preschool classroom, resource room, children with moderate to severe autism classroom, classroom for children with autism and higher academic skills of a public semirural elementary school | - Assessment of behaviour function: observations (1 week), Functional Assessment Interview Form (FAI; O’Neill et al., 1997) and function matrix (Umbreit et al., 2007) (both completed during in-service 1 day 6-hour training), Building a Support Plan form including competing pathways summary (O’Neill et al., 1997), Functional Analysis (function: for Susan-attention of teacher, for Karla, Michael and Jack-escape) - Implementation of multicomponent intervention: Functional Communication Training, DRA for 1^st^ replacement behaviour, DRI for 2^nd^ replacement behaviour, prompting hierarchy, reinforcement, environmental redesign, extinction, token economy, skills teaching via Discrete Trial Teaching, photograph communication system to request break.   (percentage accuracy of implementation by teachers assessed) | ***Values:***  V1, V2, V3  ***Systems:***  S1, S2  ***Science & Technologies:***  S&T1, S&T2, S&T3, S&T4  9 characteristics | 1. The problem behaviours decreased for Susan, Karla, Michael and Jack from baseline (*M* =3, range 1-8; *M* =52.2%, range 0-90%; stable at midlevel; *M* = 65.0%, range 30%-100%, respectively) to the function-based intervention phase (teacher baseline) (*M*=0; *M* = 10.0%, range 0%- 30%; *M* = 19%, range 10%-25%; *M* = 36.0%, range 10%-50%, respectively) with zero levels during maintenance for Susan, Michael and Karla, and low levels for Jack (10% of intervals). 2. Primary replacement behaviour (appropriate use of materials, compliance, on-task behaviour) increased from baseline to function-based intervention for all students. 3. Secondary replacement behaviour (functional communication) increased for Michael, but it remained at zero or near zero levels for Jack and Karla, respectively. |
| Bloom et al. (2013)  (USA) | ABAB reversal design to investigate:  1. Challenging behaviours (mouthing) rate (Responses per minute with mouthing)  2.Replacement behaviour (percentage intervals of appropriate item interaction) | 1 male (4 years old) eligible (Alejandro) with autism (ASD) in a separate room at the  school or an area in the classroom partitioned away from the rest of the classroom at a university-based preschool for children with ASD | - Assessment of behaviour function: Trial-based Functional Analysis (1 hour and 2 minutes) conducted by teachers, family was consulted for preferred objects of student, multiple stimulus without replacement (DeLeon & Iwata, 1996) preference assessment was conducted (function: automatic) - Implementation of noncontingent reinforcement (NCR: automatic reinforcement) intervention with competing objects | ***Values:***  V1, V2, V3  ***Systems:***  S1, S2  ***Science & Technologies:***  S&T1, S&T2, S&T3  8 characteristics | 1. Mouthing reduced from moderate to high (*M*= 3.31; range 0.5-5.25) in baseline to near zero (range 0-0.7) in second NCR phase. 2. Percentage of engaging in replacement behaviour was at 100% during first NCR phase and 90%-100% during the second NCR phase. |
|  |  |  |  |  | *(Continued)* |
| **Table S2.** *(Continued)* | | | | | |
| Authors (Year)(Country) | Design & Outcome Measures | Participants (CYP) & Setting | Intervention | PBS Framework Components | Outcome |
| Butler & Luiselli (2007)  (USA) | ABAB reversal design to investigate occurrences of challenging behaviours (percentage of occurrences of CB) | 1 female (14 years old) with autism (ASD) in a partitioned section of the classroom at a private school for children with developmental disabilities | - Assessment of behaviour function: Functional Analysis (across 9 days in 10-minute sessions)-three phases: 1) 4 conditions programmed in a multielement design (Iwata et al., 1994), 2) evaluation whether CB was influenced by three different instructional requests, 3) five instructors presented Request 1 directives in the same manner as in Phase 2 (function: escape) - Implementation of noncontingent reinforcement (NCR: escape) and instructional fading intervention | ***Values:***  V1, V2, V3  ***Systems:***  S1  ***Science & Technologies:***  S&T1, S&T2, S&T3  7 characteristics | CB reduced from *M*= 59% per session in baseline to near zero occurrence when intervention was reintroduced (*M* = 2%) and task requests continued to be increased |
| Calloway & Simpson (1998)  (USA) | ABA reversal design to investigate rate of challenging behaviours (frequency count of CB) | 1 male (4 years old; cognitive delay) (Jarrett), and 1 male (3 years old; suspected autism and developmental delay) (Terrance) in early childhood special education classroom at community or public school preschool in a midwestern city | - Assessment of behaviour function: Direct observations, Motivation Assessment Scale adapted for use by early childhood interventionists (MAS; Durand & Crimmins, 1992) completed by teacher for Jarret and by teacher and senior author for Terrance, informal assessment (function: Jarrett-attention, Terrance-escape) - Implementation of noncontingent social attention (NCR: attention) and extinction for Jarrett, and environmental modifications (verbal prompts, reminders, and visual schedule) for Terrance | ***Values:***  V1, V2, V3  ***Systems:***  S1  ***Science & Technologies:***  S&T1, S&T2, S&T3, S&T4  8 characteristics | CB reduced for all students, for Jarrett from *M*=43 aggressive acts per day in baseline to 4 during intervention, and for Terrance from *M*= 30 times of wandering behaviour per session in baseline to 7 times per session during intervention (with an increasing trend in reversal phase for both) |
|  |  |  |  |  | *(Continued)* |
| **Table S2*.*** *(Continued)* | | | | | |
| Authors (Year)(Country) | Design & Outcome Measures | Participants (CYP) & Setting | Intervention | PBS Framework Components | Outcome |
| Cavalari et al. (2014)  (USA) | ABABC reversal design to investigate challenging behaviour (episodic frequency of skin picking) | 1 female (17 years old) with autism (ASD) in a classroom at a specialised school for children and youth who had autism and related developmental disabilities | - Assessment of behaviour function: Pre-intervention assessment with mother, classroom teacher completed Functional Analysis Informant Record for Teachers (FAIR-T; Edwards, 2002), student responded to self-report survey, adapted from the Behavior Interview and Reinforcement Survey (Solution Tree, 2009), all classroom staff completed the Functional Assessment Observation Form (O’Neill et al., 1997), direct observation (function: escape and automatic) - Implementation of differential reinforcement and competing response training (DRA/I) for simplified habit reversal, reminding behavioural expectations, verbal prompts, tiered token economy system, visual cues | ***Values:***  V1, V2, V3  ***Systems:***  S1, S2  ***Science & Technologies:***  S&T1, S&T2, S&T3, S&T4  9 characteristics | Skin picking reduced from a range of 0-22 episodes per day in baseline to 0-5 episodes per day during the intervention second implementation (during treatment fading reduced further to 0-4 episodes per day). |
| Cihak & Gama (2008)  (USA) | A changing criterion with an imbedded reversal design to investigate:  1. Challenging behaviours rate (percentage of intervals with CB)  2. Alternative behaviour rate (percentage of intervals with task engagement) | 2 females (12 and 13 years old) and 1 male (13 years old) with moderate to severe mental retardation (Intellectual Disability) in middle school resource classrooms (of each student) | - Assessment of behaviour function: Brief Functional Analysis (BFA) with conditions (10 min each) similar to Iwata, Dorsey, Slifer, Bauman, and Richman (1982/1994) and Northup et al. (1991), semi-structured interview (O'Neill et al., 1997) with teachers (ABC data, reinforcers assessment), confirmatory analysis after BFA (function: escape) - Implementation of noncontingent escape access to self-reinforcement (NCR: escape) as a student-directed intervention after training of cards use to request breaks, fading cards, timer to remind break ending, prompting hierarchy, self-reinforcement, task analysis, replacement behaviours reinforced | ***Values:***  V1, V2, V3  ***Systems:***  S1  ***Science & Technologies:***  S&T1, S&T2, S&T3, S&T4  8 characteristics | 1. Challenging behaviours mean percentage of intervals decreased for Adele, Barron and Camilla from the escape (53.6%, 70.6%, 76.3%, respectively), attention (6.5%, 10%, 16.5%, respectively) and control (5%, 0%, 2%, respectively) conditions to the final phase of the intervention (1 card) (0.7%, 2.3%, 3.6%, respectively). 2. Task engagement increased for Adele, Barron, and Camilla. |
|  |  |  |  |  | *(Continued)* |
| **Table S2*.*** *(Continued)* | | | | | |
| Authors (Year)(Country) | Design & Outcome Measures | Participants (CYP) & Setting | Intervention | PBS Framework Components | Outcome |
| Clarke & Duda (2019)  (USA) | ABAB reversal design to investigate:  1.Challenging behaviour rate (Percentage of intervals with CB)  2.Engagement rate (Percentage of intervals with engagement)  3.Positive affect rate (Percentage of intervals with positive affect) and quality of life indicators | 1 female (13 years old) with multiple disabilities including autism (ASD) in a special education classroom at a public middle school (intervention in various areas of school during PE routine with her special education classmates e.g. transition to PE track, track, locker room, classroom after PE) | - Assessment of behaviour function: over 4 weeks PBS facilitator conducted direct observation (10 consecutive days), interviews with adults (teacher, teacher aide, PE coach, mother) and peer buddies using Functional Assessment Interview (FAI) (O’Neill et al., 1997), review of IEP of previous school year (6^th^ grade) (function: escape from activities difficult unpredictable and nonpreferred) - Implementation of peer mediated multicomponent intervention: environmental adjustments (distractions minimized), picture schedule, PE task analysis, prompts, preferred activity for transition, reinforcement, choice of toy and class activity after PE, verbal acknowledgement of Mia’s actions | ***Values:***  V1, V2, V3  ***Systems:***  S1, S2  ***Science & Technologies:***  S&T1, S&T2, S&T3, S&T4  9 characteristics | 1. Challenging behaviour reduced from a mean of 69% (range 43–93%) of intervals across all activities during baseline and withdrawal probes to a mean of 13% (range 3–43%) during intervention sessions. 2. Engagement and positive affect increased from baseline and withdrawal sessions to intervention and generalization sessions. Additionally, perceived (by peer buddies) quality of life indicators ratings increased after the intervention. |
| Dunlap et al. (1995)  (USA) | Reversal design ABABA with a brief probe inserted during the second functional outcome condition for Jill, to investigate:  1.Challenging behaviour rate (Percentage of intervals with CB)  2.On-task behaviour (Percentage of intervals with on-task behaviour)  3.Happiness and interest (0-5 Likert scale) | 1 female (13 years old) eligible (Jill) with multiple disabilities including severe emotional disturbance, mild mental retardation, schizophrenia,  and attention deficit disorder enrolled in classroom for students with severe emotional disturbance and individualised activities in separate room with aide (typical routine context) | - Assessment of behaviour function: Functional assessment had already been conducted in Dunlap et al., (1991) (standardized instruments, direct observations, rating scales, and an 11-item questionnaire administered to 28 respondents who had regular contact with Jill over the previous few years), additionally conducted in this study direct observations and teacher interviews to assess tasks (Possible function: escape from difficult tasks) - Implementation of curricular modifications intervention (modifying written tasks to produce functional outcomes making them less aversive and providing help upon request) | ***Values:***  V1, V2, V3  ***Systems:***  S1  ***Science & Technologies:***  S&T1, S&T2, S&T3  7 characteristics | 1. Jill’s problem behaviour decreased from baseline and withdrawal (standard outcome) conditions to intervention (functional outcome) conditions. Numerical data depicted in Figure 2 of the paper. 2. On task behaviour increased during functional outcome conditions (intervention). 3. The data regarding Jill's affect demonstrated positive results with increases in her happiness and interest. |
|  |  |  |  |  | *(Continued)* |
| **Table S2.** *(Continued)* | | | | | |
| Authors (Year)(Country) | Design & Outcome Measures | Participants (CYP) & Setting | Intervention | PBS Framework Components | Outcome |
| Ellingson et al. (2000)  (USA) | Brief reversal design ABACBC to investigate challenging behaviours change (frequency of pounding for Christine and number of instances of aggression for Dereck) | 1 female (Christine: 19 years old) and 1 male (Dereck: 18 years old) eligible with severe mental retardation (Intellectual Disability) and mild cerebral palsy (Christine), and profound mental retardation (Intellectual Disability) and Angelman Syndrome (Dereck), in special education classrooms | - Assessment of behaviour function: behavioural interview in questionnaire format completed by teacher and PhD Behaviour Analyst (2^nd^ author) who also completed a confidence rating on a 7-point Likert scale, behavioural interview (same as questionnaire but also had follow-up questions) administrated to the same teacher by 1^st^ author, direct observations (4 x 30 minutes) using ABC Observation checklist form completed by teachers and 2 Research Assistants with stopwatch (function: attention for all students) - Implementation of intervention: Noncontingent attention (NCR: attention), DRA and extinction | ***Values:***  V1, V2, V3  ***Systems:***  S1, S2  ***Science & Technologies:***  S&T1, S&T2, S&T3  8 characteristics | CB decreased for Christine and Dereck from *M*=13 frequency of pounding in baseline to *M* = 2 during functional intervention with eventual elimination of CB, and from *M* = 41.7 instances of aggression in baseline to *M* = 2.3 during functional intervention with eventual *M* = 7.6 number of instances, respectively.  (Non-functional intervention demonstrated a mean of 16 during first implementation and 10.5 during subsequent implementation for Christine and a mean of 13.7 during first implementation and 8.7 during subsequent implementation for Dereck.) |
| Flynn & Lo (2016)  (USA) | Delayed multiple baseline design across students, and multiple probe design across teacher–student triads for teacher procedural integrity, to investigate:  1.Challenging behaviour (number of behaviour occurrences)  2.Replacement behaviour (number of occurrences) | 5 males (1A-12 years old, 1B-12 years old, 2A-11 years old, 2B-11 years old, 3A-11 years old) with autism (ASD) eligible (2 students for each teacher and students B for generalization purposes) in 3 special education settings (2 self-contained settings and 1 resource room) in a middle school at a large southeastern urban school district | - Assessment of behaviour function: Trial-Based Functional Analysis (TBFA) using multielement design (Kazdin, 2010) (function: 1A-Escape, 2A-Attention, 3A-Automatic reinforcement, 1B-Attention, 2B-Escape) - Implementation of intervention: DRA, extinction, response interruption & redirection, prompting verbally or with gestures, teaching manding, use of picture symbols for manding | ***Values:***  V1, V2, V3  ***Systems:***  S1, S2  ***Science & Technologies:***  S&T1, S&T2, S&T3, S&T4  9 characteristics | 1. Challenging behaviour of all students (means of 28.6, 25.6, and 23.6 occurrences for Students 1A, 2A, and 3A, respectively) during baseline reduced during DRA with feedback condition. During generalization, measures of behaviour for Students B (Figure 3) showed similar patterns as those in Students A, meaning that Students B’s challenging behaviour decreased after the teachers completed five sessions of DRA implementation with feedback.  2.Replacement behaviour occurrences increased from no occurrence in baseline for students of group A to considerable increases during DRA (Figure 3). Generalisation measure of behavioural changes in Students B (Figure 3) showed similar patterns of increase. |
|  |  |  |  |  | *(Continued)* |
| **Table S2*.*** *(Continued)* | | | | | |
| Authors (Year)(Country) | Design & Outcome Measures | Participants (CYP) & Setting | Intervention | PBS Framework Components | Outcome |
| Foran et al. (2015)  (UK) | Case series for study 2 (Cian: ABC and Lucy: AB) measuring challenging behaviour changes (percentage of interval occurrences with CB) | 2 case studies, 1 male and 1 female in resource room and special classroom after reintegration (Cian), and either special classroom or resource room (Lucy) respectively at special school | - Assessment of behaviour function: Functional Analysis (function: Cian-escape from difficult academic activities; Lucy-attention and escape) - Implementation of intervention: Cian- request break (DRA), token economy for first intervention and Achieve! Programme and individual ABA-based teaching program for second intervention; Lucy- redirecting to alternatives (DRA), time-based reinforcement, rules explained at session beginning | ***Values:***  V1, V2, V3  ***Systems:***  S1, S2  ***Science & Technologies:***  S&T1, S&T2, S&T3, S&T4  9 characteristics | Challenging behaviours reduced for both Cian (from 18% of intervals to average 5.25% of intervals between baseline and first Intervention decreasing and remaining 2 with 2 exceptions over a nine- month period) and Lucy (Figure 5 of the study depicted numerical data from baseline to April and from April to June)  Additional outcomes: Social inclusion (Cian reintegrated in special classroom and Lucy in mainstream classroom but no formal measures) |
| Friedman & Luiselli (2008)  (USA) | ABAB reversal design to investigate daytime sleep (cumulative number of hours sleeping) | 1 male (13 years old) with Autism (ASD) in a classroom and other school settings (like corridors, lunch and snack area) at a specialised school | - Assessment of behaviour function: Motivation Assessment Scale (MAS) (Durand & Crimmins, 1988), Functional Analysis Screening Tool (FAST) scale, classroom staff completed A-B-C recording checklist (Bijou, Peterson, & Ault, 1968)(function: primarily automatic and may also serve escape) - Implementation of multicomponent intervention: stimulus change (environmental redesign), Response Interruption and Redirection, positive reinforcement and DRA. | ***Values:***  V1, V2, V3  ***Systems:***  S1, S2  ***Science & Technologies:***  S&T1, S&T2, S&T3  8 characteristics | Daytime sleep reduced from average 46 minutes in baseline and 22 minutes in reversal to non-occurrence of sleeping during interventions. During follow up, daytime sleepiness was eliminated to zero rates. |
| Hansen & Wadsworth (2015)  (USA) | Reversal design ABCDEAEF to investigate:  1.Eye pocking (duration in seconds per session)  2.Hand clapping (frequency) | 1 male (10 years old) with autism, intellectual disability, hearing impairment, and astigmatism in a self-contained special education classroom | - Assessment of behaviour function: Interview with teacher based on Functional Assessment Checklist for Teachers and Staff (FACTS; March et al., 2000), descriptive analysis (1-hour antecedent-behaviour-consequence observation during instructional period and class-wide instruction), hypothesis test (baseline)-ignore condition (function: automatic) - Implementation of antecedent intervention: providing choice and environmental enrichment, glasses started being used during study and student had cochlear implant already before study started | ***Values:***  V1, V2, V3  ***Systems:***  S1  ***Science & Technologies:***  S&T1, S&T2, S&T3, S&T4  8 characteristics | 1. Eye pocking reduced from *M*=57.71 seconds duration per session (range 0 - 180) to *M*=1 second of eye poking (range 0- 5) at the end of the intervention. 2. Hand clapping reduced from rate of 20.14 times per session (range 4-36) in baseline to *M*=0.6 claps (range 0-1) at the end of the intervention. At follow-up, there was *M*=1 clap (range 0-2). |
|  |  |  |  |  | *(Continued)* |
| **Table S2*.*** *(Continued)* | | | | | |
| Authors (Year)(Country) | Design & Outcome Measures | Participants (CYP) & Setting | Intervention | PBS Framework Components | Outcome |
| Karsh et al. (1995)  (USA) | Multiple baseline across participants (passive response as control condition for Adam and Susan, and demands other to toileting and demand absence as control conditions for Brad) to investigate:  1.Challenging behaviours (percentage of time engaged in problem behaviours)  2.Compliance (percentage of time engaged in compliance) | 2 males (Adam: 11 years old; Brad: 7 years old) and 1 female (Susan: 11 years old) with developmental delay, failure to thrive, cerebral palsy, dysarthria, severe mental retardation and seizure disorder (Susan); autism, severe mental retardation and possible seizure disorder (Adam); developmental disability, severe mental retardation and seizure disorder (Brad), in 3 classrooms for CYP with disabilities | - Assessment of behaviour function: Interview with staff following format by O'Neill, Homer, Albin, Storey, and Sprague (1990), direct observations (2-5 hours) using observation form of O'Neill et al. which guided recording of occurrence of behaviours and the conditions of occurrence (and non-occurrence) (function: escape for all students) - Implementation of multicomponent intervention: modifications of tasks including modified length of tasks, task analysis, more detailed instructions, positive reinforcement, prompting hierarchy, modelling for skills building, use of signs and pictures to give instructions and cue breaks, one student was also receiving drug therapy | ***Values:***  V1, V2, V3  ***Systems:***  S1  ***Science & Technologies:***  S&T1, S&T2, S&T3, S&T4  8 characteristics | 1. Reductions of time engaged in CB for Adam (for active tasks), Susan (for active tasks), and Brad (for toileting) from baseline (75%, 67%, 75% respectively) to intervention (8%, 14%, 5% respectively) with 0% during follow-up as depicted in graphs. 2. Increases of time engaged in compliance from baseline to intervention were reported for Adam and Susan for active tasks, and for Brad for toileting. |
| Lalli et al. (1993)  (USA) | Combination multiple baseline across students and a component analysis to investigate:  1.Challenging behaviours (number per  minute and intervals for problem behaviours)  2. Adaptive behaviours (number per session for target responses) | 1 male (Bob; 10 years old) with Down Syndrome, 1 female (Mary; 14 years old) and 1 male (Al; 10 years old) with cerebral palsy and Intellectual Disability (reported that teachers work with CYP with mental retardation) in special classrooms | - Assessment of behaviour function: Problem-identification interview with teachers, scatter plot analysis over a 5-day period to identify the times when target behaviours occurred, narrative recordings (3 x 2hours observations) and descriptive analysis (5x 1h observations per student) (function: attention, for Al escape as well) - Implementation of multicomponent intervention: Differential Reinforcement of Alternative behaviour (DRA), extinction, and adaptive skills teaching/training (teaching to mand), providing choice and use of picture booklet for Al. | ***Values:***  V1, V2, V3  ***Systems:***  S1, S2  ***Science & Technologies:***  S&T1, S&T2, S&T3, S&T4   1. characteristics | - 1. Challenging behaviours of Bob (aggression), Mary and Al (self-injury) decreased from baseline (*M* = 3.3 aggressions per minute, *M* = 57% of intervals, *M*= 6 responses respectively) to intervention (*M*= 0.4 aggressions per minute, *M*= 10% of intervals, *M*= 1.4 responses). Low levels were maintained when intervention was discontinued in the next phase (*M* = 0.6, *M*= 2%, *M*= 0 respectively).   2. Adaptive skills training resulted in increase of the target adaptive behaviours for Al, Bob and Mary. |
|  |  |  |  |  | *(Continued)* |
| **Table S2.** *(Continued)* | | | | | |
| Authors (Year)(Country) | Design & Outcome Measures | Participants (CYP) & Setting | Intervention | PBS Framework Components | Outcome |
| Lane et al. (2006)  (USA) | ABCBAB reversal design to investigate:  1.Skin picking rate (Percentage of time with skin picking)  2.Replacement behaviour rate (Percentage of time with occupied hands) | 1 male (9 years old) with Attention Deficit Hyperactivity Disorder (ADHD), learning disability, a speech-language impairment, and a full-scale IQ of 77 in a classroom at a self-contained public school | - Assessment of behaviour function: Teacher functional assessment interview and direct observation (ABC data collected for 2 hours in multiple settings). It was hypothesized that manipulatives during reading instruction would decrease skin picking (function: automatic) - Implementation of intervention: Competing activities (explained to the participant), implemented by providing a box with three objects and choice of competing activity to occupy hands, medication was in use | ***Values:***  V1, V2, V3  ***Systems:***  S1  ***Science & Technologies:***  S&T1, S&T2, S&T3, S&T4  8 characteristics | 1. Skin picking reduced from *M*= 67% in baseline to *M*= 8% during the first intervention, showed increasing trend in phase C and once medication was resumed it reduced again. On Day 18 the intervention was breached. From Day 19 to 23 intervention resumed and skin picking reduced. During reversal skin picking increased to *M*= 74% and decreased again when intervention was implemented. Intervention ended prematurely when Jason changed schools. 2. Replacement behaviour rate increased. |
| Lang et al. (2010)  (USA) | Alternating treatments design to investigate elopement (percentage of intervals with elopement) | 1 male (4 years old) with Asperger’s syndrome (ASD) in a typical classroom with other children with developmental delay and resource room (for DTT) | - Assessment of behaviour function: Functional Analysis (conditions were alternated according to a multielement design in each setting), influence of setting was examined systematically using an ABAB design (A represented the resource room and B represented the classroom) (function: resource room-attention, classroom access to tangibles) - Implementation of Noncontingent reinforcement (NCR) and environment adjustments | ***Values:***  V1, V2, V3  ***Systems:***  S1  ***Science & Technologies:***  S&T1, S&T2, S&T3  7 characteristics | The function-based intervention resulted in lower levels of elopement in each setting. In the resource room, where elopement was maintained by attention, the attention-based intervention resulted in lower levels of elopement than the tangible-based intervention. In the classroom, where elopement was maintained by access to a DVD, the tangible-based intervention resulted in lower levels of elopement. (Figure 2 of the primary study depicts the percentage of intervals with elopement) |
| Larkin et al. (2016)  (USA) | ABAB reversal design to investigate:  1.Challenging behaviours (CB) (percentage of intervals with disruptive behaviour)  2. Engagement (level of engagement) | 3 males (Lenny: 7 years old; Ben: 4 years old; Derrick: 5 years old) with autism (ASD) in various educational settings including classrooms at a special educational centre | - Assessment of behaviour function: Interviews with teachers using Functional Assessment Checklist for Teachers and Staff (FACTS; March et al., 2000), Trial-based Functional Analysis based on Sigafoos and Saggers (1995) and Bloom et al. (2011) procedures (40 trials in total up to 60 seconds each across 4–10 days) (function: Lenny- attention and escape, Ben and Derrick-tangibles) - Implementation of intervention: DRA, DRO, Extinction, social stories with pictures explaining the interventions, token boards, additional supports already in place (visual schedules, class-wide positive behaviour supports and token board) | ***Values:***  V1, V2, V3  ***Systems:***  S1, S2  ***Science & Technologies:***  S&T1, S&T2, S&T3, S&T4  9 characteristics | 1. Challenging behaviours of Lenny, Ben, and Derrick reduced from baseline (*M* = 30.78 and *SD* = 22.42, *M* = 29.04 and *SD* = 11.16, *M* = 26.11 and *SD* = 14.61, respectively) to final intervention phase (*M* = 3.47 and *SD* = 5.37, *M* = 8.2 and *SD* = 10.26, stable near-zero levels, respectively). 2. Engagement increased after the intervention. |
|  |  |  |  |  | *(Continued)* |
| **Table S2*.*** *(Continued)* | | | | | |
| Authors (Year)(Country) | Design & Outcome Measures | Participants (CYP) & Setting | Intervention | PBS Framework Components | Outcome |
| Lohrmann-O'Rourke & Yurman (2001)  (USA) | ABAB reversal design to investigate challenging behaviour (percentage of intervals with mouthing) | 1 male (6 years 3 months old) with Down syndrome, seizure disorder, severe mental retardation in a self-contained K-3 classroom with other children with disabilities at local school | - Assessment of behaviour function: Interview of teaching team, mother reports on antibiotics administration, observations (multielement manipulation in 5 settings, 5-minute sessions) for Functional Analysis, brief preference assessment (function: multiple functions- circle: attention, alone: CB alternative to no option, task demand: escape; sinus infection as Motivating Operation) - Implementation of intervention: Choices (non-disruptive and based on the preference assessment) provided, materials added to enhance options, antibiotics received for sinus infection (MO) | ***Values:***  V1, V2, V3  ***Systems:***  S1  ***Science & Technologies:***  S&T1, S&T2, S&T3, S&T4  8 characteristics | Decreased occurrences of mouthing were observed in both intervention phases for all three routines, from mean occurrence across conditions of 61% when the infection was present and 40.5% when the infection cleared up during baseline, to 34% and 16.5%, respectively during intervention. |
| Moore et al. (2009)  (USA) | Quasi-longitudinal (quasi-experimental) descriptive case study, ABA reversal for intervention to investigate challenging behaviours occurrences (trials completed without CB for intervention evaluation) | 1 male (12 years old) with cerebral palsy, global developmental delay, and spastic quadriplegia in a special education classroom of a middle school | - Assessment of behaviour function: Functional Analyses (FA) were presented in quasi-random order using a multielement design (Iwata, Dorsey, Slifer, Bauman, & Richman, 1994). For study 2, FA were conducted by classroom aide (after coaching from author) on six occasions over approximately 6 months (M= 1 session per month; range 6-9 sessions per day (function: escape) - Implementation of intervention: treatment consistent with Functional Communication Training (1. choice for task engagement or avoidance, 2. identification of preferred activity reinforcer for task completion, 3. visual signal of reinforcement availability, and 4. faded-in delays to reinforcement), medication was already in use. | ***Values:***  V1, V2, V3  ***Systems:***  S1, S2  ***Science & Technologies:***  S&T1, S&T2, S&T3, S&T4  9 characteristics | Trials completed without challenging behaviour increased, thus occurrences of challenging behaviour decreased as a result of the intervention implementation. No problem behaviour was observed during treatment sessions (phases A), and Alex responded with "Yes" to the choice of engaging in the math problem task in 100% of trials, completing them all successfully. During reversal phase (phase B), challenging behaviour was observed in 2 of 3 three-trial sessions, and Alex refused to participate in one of the trials. |
| Mueller & Kafka (2006)  (USA) | A reversal design with a multi-element comparison phase (ABAC & follow-up) to investigate challenging behaviour rate (percentage of intervals with object mouthing) | 1 female (4 years old) with significant developmental delay (SDD) in a special education needs preschool classroom (designated area in classroom) of a public school | - Assessment of function: parent and teacher interviews, observations (Bijou, Peterson & Ault, 1968) (2 x 3 weeks), free operant (2x10 minute) and paired choice preference assessments (1x10 minute) Functional Analysis (function: automatic) - Implementation of noncontingent reinforcement (NCR: automatic) with fixed time schedules of food delivery 20, 30 or 60 seconds, and during follow-up additionally response blocking | ***Values:***  V1, V2, V3  ***Systems:***  S1, S2  ***Science & Technologies:***  S&T1, S&T2, S&T3  8 characteristics | Figure 6 shows the results. After the initial baseline, FT 20- and FT 60-seconds treatments were compared in a multi-element phase. After deciding that FT20 was more effective, a second baseline was presented. The FT20 was then implemented on its own. After a few sessions with decreased levels of mouthing the schedule was faded to FT 30 seconds. Object mouthing during follow-up was eliminated but response-blocking was also used infrequently. |
|  |  |  |  |  | *(Continued)* |
| **Table S2*.*** *(Continued)* | | | | | |
| Authors (Year)(Country) | Design & Outcome Measures | Participants (CYP) & Setting | Intervention | PBS Framework Components | Outcome |
| Mueller & Nkosi (2007)  (USA) | ABAB reversal design for analog treatment and multiple baseline across 3 professionals for intervention in classroom to investigate challenging behaviours (self-injurious-SIB and aggression) (percentage of interval with CB) | 1 female (15 years old) eligible with profound mental retardation and autism (ASD) in a special education classroom and work room | - Assessment of behaviour function: Record review (e.g., teacher journals, IEPs, teacher-collected behavioural data from classroom, psychological assessments), teacher interviews using The Functional Assessment Informant Record for Teachers (Edwards, 2002; Doggett, et al., 2002), functional assessment rating scales (e.g., Motivation Assessment Scale, MAS; Durand & Crimmins, 1988), direct observations in form of narrative descriptive observations and ABC observations (Bijou, et. al., 1968), Functional Analysis (5-minutes conditions in a small office within classroom), based on the ones described by Iwata, et al. (1982/1994), conducted by behaviour consultant (function: escape) - Implementation of Differential Reinforcement of Alternative (DRA), extinction, prompting, and choice of reinforcer to lessen aversiveness of tasks | ***Values:***  V1, V2, V3  ***Systems:***  S1, S2  ***Science & Technologies:***  S&T1, S&T2, S&T3  8 characteristics | A significant reduction of aggression and SIB across teachers in the natural classroom environment was reported after the baseline when there was a high frequency of aggression and SIB. Figure 7 (analog condition) and figure 8 (classroom condition) present the outcomes visual data in the study for Meredith. |
| Myles & Hirsch (1996)  (USA) | ABAB reversal design to investigate challenging behaviour rate (event recording for pica attempts) | 1 female (10 years and 3 months old) with autism (ASD) and pica in a self-contained special education classroom for students with autism or behaviour and/or communication disorders at a public school. | - Assessment of behaviour function: Functional Analysis showed that pica function was not to escape tasks, to gain attention, or to earn tangibles (function: concluded automatic reinforcement) - Implementation of intervention: Response interruption and redirection, verbal reminders to use pica box, choice of items, pica box offered in the morning noncontingently and during the day as an alternative to challenging behaviour. Additionally, medication was already in use. | ***Values:***  V1, V2, V3  ***Systems:***  S1  ***Science & Technologies:***  S&T1, S&T2, S&T3, S&T4  8 characteristics | Mean occurrence of pica attempts reduced from 13.67 (range = 6 to 22) in baseline and a mean of 11.00 times (range = 5 to 16) in reversal, to a mean of 4.40 attempts (range = 1 to 7) and a mean of 3.89 (range = 1 to 10) during the first and second implementation of the intervention respectively. |
|  |  |  |  |  | *(Continued)* |
|  |  |  |  |  |  |
| **Table S2*.*** *(Continued)* | | | | | |
| Authors (Year)(Country) | Design & Outcome Measures | Participants (CYP) & Setting | Intervention | PBS Framework Components | Outcome |
| Paris et al. (2019)  (UK) | Single subject design with multicomponent interventions (baseline, 1^st^ and 2^nd^ intervention, maintenance) to investigate:  1.Challenging behaviour rate and scores (percentage of daily partial interval average; pre- and post- intervention scores in Behavior Problems Inventory for Individuals with Intellectual Disabilities-Short Form (BPI-S; Rojahn et al., 2012))  2. Adaptive behaviour scores using the Vineland Adaptive Behavior Scales – Teacher Rating Form (VABS) (Sparrow, Balla & Cicchetti 1984) | 1 female (Lisa: 16 years and 10 months old) and 2 males (Andy: 14 years and 5 months old; Fred: 13 years and 3 months old) in a classroom at a special school for students with severe Intellectual Disabilities | - Assessment of behaviour function: Motivation Assessment Scale (MAS; Durand & Crimmins, 1992) and Questions about Behavioral Function (QaBF; Matson et al, 1996), review of quantitative and qualitative records from student files, ABC charts (in classroom, break and lunch) completed during direct observations (at minimum 5 x 1 hour for each participant), then summary of data and creation of Behaviour Support Plans (function: Andy- escape & access to tangibles; Lisa- escape & attention of adult; Fred-escape & access to tangibles; additionally, for Fred challenging behaviour appeared to occur more during transitions from high- to low-preferred activities and during unstructured times and for Andy during transitions from a preferred to a non-preferred activity) - Implementation of multicomponent interventions: Visual schedule board (VSB), Differential Reinforcement of Alternative behaviour (DRA), Differential Reinforcement of Low and zero rates of behaviour (DRL) and use of token economy, Functional Communication Training (FCT). Additionally, use of PECS, Makaton, and other evidence-based approaches such as hearing aids was in place at the school. | ***Values:***  V1, V2, V3  ***Systems:***  S1, S2  ***Science & Technologies:***  S&T1, S&T2, S&T3, S&T4   1. characteristics | 1. Challenging behaviours of Fred, Lisa and Andy decreased during the intervention phases, and zero rates started being recorded across the day after 5, 6 and 15 sessions, respectively.   BPI-S aggressive/destructive behaviour scores for all students were lower at follow-up. Specifically, scores for Lisa reduced from 483 to 246, for Andy reduced from 105 to 28 and for Fred reduced from 12 to 3. No change in the BPI-S stereotypy scores for Fred or Lisa, but there was a slight increase in the stereotypy scores for Andy from 34 to 43. BPI-S self-injurious behaviour scores increased slightly for Andy from 2 to 9, while scores for Fred and Lisa decreased from 52 to 35 and from 6 to 1, respectively.   1. The VABS composite scores remained stable for Fred but increased slightly for Lisa and Andy. |
| Pennington et al. (2012)  (USA) | ABA reversal design to investigate challenging behaviour e.g. elopement (percentage of intervals in which elopement occurred) | 1 male (7 years old) with autism (ASD) in a self-contained special education classroom | - Assessment of behaviour function: Functional Assessment Screening Tool (FAST; Iwata, 1995) for interviews, observations, and narrative antecedent-behaviour-consequence data (function: attention) - Implementation of intervention: Differential Reinforcement of Alternative behaviour (DRA) with a variable interval schedule of reinforcement (40 seconds) and extinction of reinforcement | ***Values:***  V1, V2, V3  ***Systems:***  S1, S2  ***Science & Technologies:***  S&T1, S&T2, S&T3   1. characteristics | 1. Challenging behaviour reduced from *M* = 50% of intervals (range 25-85%) during baseline to *M* = 14% (range 0-28%) during intervention when teacher delivered verbal attention contingent on appropriate behaviour (DRA) and increased during withdrawal phase to *M* = 18.5% (range 0-45%). 2. Appropriate behaviours increased (hand raising, question answering, orienting towards teacher, looking at teacher, staying seated, participation) (informal data based on observations and reports). |
|  |  |  |  |  | (Continued) |
| **Table S2.** *(Continued)* | | | | | |
| Authors (Year)(Country) | Design & Outcome Measures | Participants (CYP) & Setting | Intervention | PBS Framework Components | Outcome |
| Pitts et al. (2019)  (UK) | Pre- and post- test within-group design to investigate:  1.Challenging behaviour using the Behavior Problems Inventory – Short Form for individuals with intellectual  Disabilities (BPI-S; Rojahn et al., 2012)  2. Adaptive behaviour: Vineland Adaptive Behavior Scale–Survey (VABS; Sparrow et al., 2005)  3.Language and learning skills: Assessment of Basic Language and Learning Skills– Revised: ABLLS-R; Partington, 2006)  4. Academic attainment using the Early Years  Foundation Stage (EYFS) Framework (DfE, 2012, 2013) and the Performance (P-scale)  Attainment Targets for Pupils with Special Educational Needs (DfE, 2014). | 16 students 3-19 years old (Early Years and Key Stage 1: 5 males, age *M*= 65.4 months; Key Stage 2: 5 males and 1 female, age *M*=108.6 months; Key stage 3: 3 females and 2 males, age *M*= 151.2 months) with autism (ASD) and associated learning difficulty (14 also had additional diagnoses, including attention deficit hyperactivity disorder, sensory processing disorder and global developmental delay) in special education classrooms for children with ASD at a special school for students with ASD and/or other disabilities | - Assessment of behaviour function: Procedures utilised were similar to the Foran et al. (2015) study which reports that Behaviour Analysts conduct Functional Analysis to determine function, use of assessments of behaviour function to design and implement Behaviour Support Plans and function-based interventions. - Implementation of function-based interventions based on function-based Behaviour Support Plans after assessments of the function of behaviour are conducted. Additionally, it was reported that students were taught appropriate replacement behaviours and functional communication. | ***Values:***  V1, V2, V3  ***Systems:***  S1, S2  ***Science & Technologies:***  S&T1, S&T2, S&T3, S&T4  9 characteristics | 1. Statistically significant reductions were reported in BPI-S composite scores (*t* (15) = –3.377, *p* < 0.005; medium effect size), specifically in self-injurious behaviour (*t* (15) = –4.058, *p* < 0.005; medium effect size) and stereotyped behaviour (*t* (15) = –11.949, *p* < 0.001; large effect size), suggesting that function-based behaviour support plans were effective in reducing challenging behaviour. Aggressive and destructive behaviour reductions reported but not statistically significant with small effect size. 2. Statistically significant gains in VABS adaptive behaviour composite scores were reported by parents and teachers. 3. Statistically significant gains in total ABLLS-R scores were reported. 4. Statistically significant gains for English, mathematics, science and PSHE were reported. Students exceeded their predicted EYFS and P-scale scores for all subjects after one academic year. |
|  |  |  |  |  | *(Continued)* |
| **Table S2.** *(Continued)* | | | | | |
| Authors (Year)(Country) | Design & Outcome Measures | Participants (CYP) & Setting | Intervention | PBS Framework Components | Outcome |
| Rispoli et al. (2018)  (USA) | ABAB reversal design to investigate the challenging behaviour of vocal scripting, a form of vocal stereotypy (percentage of intervals with CB) | 3 males (Tommy and Herbert:10 years old, Elvis: 7 years old) with autism (ASD) in a private educational centre for children with ASD implementing ABA (Tommy and Elvis: in cubicles of an open room, Herbert in a private room) | - Assessment of behaviour function: Motivation Assessment Scale (MAS; Durand & Crimmins, 1992), direct observation using the Functional Assessment Observation Form (FAOF; Rispoli et al., O’Neill et al., 1997), Trial-Based Functional Analysis (TBFA) planning worksheet (Rispoli, Neely, Healy, & Gregori, 2016) to guide planning, TBFA (Bloom et al., 2011) and a modified version of the TBFA (function: automatic reinforcement and possibly attention; attention as an abolishing operation) - Implementation of noncontingent reinforcement (NCR: attention) (cubicles in the room to minimise distractions already in place) | ***Values:***  V1, V2, V3  ***Systems:***  S1, S2  ***Science & Technologies:***  S&T1, S&T2, S&T3  8 characteristics | Challenging behaviours of Herbert, Tommy and Elvis decreased from baseline (*M* = 82% and range = 65%-97%, *M* = 63% and range =53%-90%, *M* = 39% and range =33%- 43%, respectively) to the final intervention phase (*M* = 6% and range= 3%-10%, *M*= 0%, *M*= 13% and range =7 %-33%, respectively). |
|  |  |  |  |  | *(Continued)* |

CB=Challenging behaviour

SIB=Self-injurious behaviour

DRA=Differential reinforcement of alternative behaviour

DRI= Differential reinforcement of incompatible behaviour

DRO= Differential reinforcement of other behaviour

NCR=Noncontingent reinforcement

FCT=Functional Communication Training

TBFA=trial-based Functional Analysis

| **Table S3.** *Detailed studies addressing RQ3* | | |
| --- | --- | --- |
| Authors (Year) (Country) | Methods | Findings |
| Artman-Meeker et al. (2017)  (USA) | Questionnaire (6 item satisfaction survey) and 4 open-ended questions completed by 3 school staff members (pre-service behaviour analysts) | - Participants were positive about the intervention reporting that Bug-In-Ear (B-I-E) coaching improved their ability to use Functional Communication Training (FCT) and that they would recommend it to others. - They also reported that B-I-E coaching was somewhat distracting, but it was less disruptive than other forms of coaching. Two participants mentioned difficulties working with more than one student during coaching phase. - Participants suggested combining B-I-E coaching with in-person debriefing for increasing collaboration with coach. |
| Banda et al. (2009)  (USA) | Likert scale brief social validity questionnaire and qualitative responses about the intervention completed by teaching assistant and mother of the child | - The mother of the child was encouraged and pleased with the outcomes and reported that the child enjoyed work more and was less resistant to work with service providers. - The teaching assistant reported that the child was calmer. |
| Banda et al. (2012)  (USA) | Modified Likert-type questionnaire, the Intervention Rating Profile-15 (IRP-15; Martens & Witt, 1982), completed by the teacher, teaching assistant, and parent. In addition, qualitative responses from the teaching assistant and parent were obtained on the importance and acceptance of the intervention strategy, ease of implementation, and its long-term effects. | - The teacher gave the intervention an average score of 5.67 (range 4–6), which indicates that the teacher strongly agreed with 11 of 15 items on the scale. - The teaching assistant gave the intervention an average score of 5.73 (range 4–6), which indicates that the teaching assistant strongly agreed with 12 of 15 items on the scale. - The parent gave the intervention an average score of 5.87 (range 5–6), which indicates that the parent strongly agreed with 13 of 15 items on the scale. - During qualitative interviews, the teaching assistant reported that the student occasionally found and held a blanket during leisure time but relinquished it upon request without self-injurious behaviour. The parent was pleased with the progress and noted collateral effects. |
| Bethune & Wood (2013)  (USA) | Likert scale social validity questionnaire (1-5 rating) and comments in open-ended section, completed by teachers, and a modified questionnaire for the school psychologist | - All four teachers strongly agreed with the three statements related to the importance, future and continued use of the function-based interventions. Furthermore, three of the teachers strongly agreed and one teacher scored a 3 for the two statements related to the effectiveness of the intervention indicating increase of adaptive and decrease of challenging behaviours. Additionally, three teachers responded and agreed strongly with the four statements related to the effectiveness, non-intrusiveness, cost-efficiency, and acceptance of the coaching procedures. - One teacher wrote the following response in the comments section: “I learned a lot from the coaching and implementing function-based interventions. I am going to try and implement these interventions in the regular education classroom. I am going to provide coaching to the regular education teacher. I am very happy with the coaching and results”. - The school psychologist agreed strongly with the statements related to the importance and effectiveness of the function-based interventions, and the effectiveness, cost-efficiency, willingness to recommend and acceptance of the coaching procedures. She rated a 4 for non- intrusiveness of the coaching procedures on the school schedule. |
| Butler & Luiselli (2007)  (USA) | Anecdotal data reported by researcher in discussion section related to how staff responded to intervention. | Researchers reported that the intervention was feasible (easily integrated into classroom activities) and considered acceptable (well-received) by staff members at the educational setting. |
|  |  | *(Continued)* |
| **Table S3.** *(Continued)* | | |
| Authors (Year) (Country) | Methods | Findings |
| Calloway & Simpson (1998)  (USA) | Anecdotal data by researchers regarding socially important goals of intervention. | The social importance of the goals chosen for each student and the rationale behind the choice of the target behaviours (e.g., absence of participation of student in classroom activities, affecting peer relationships) were reported informally. |
| Cavalari et al. (2014)  (USA) | Anecdotal data reported by researcher in discussion section related to staff experiences with the intervention. | - Infrequent skin picking during follow-up suggesting durable results of the intervention. - Stakeholder participation, expertise of staff and regular school attendance of student promoted intervention consistency and fidelity but there was no generalization to the home setting. |
| Cihak & Gama (2008)  (USA) | Intervention Rating Profile-15 (IRP-15; 16 items and a 6- point Likert scale) completed by teachers. | Teachers strongly agreed with the following as stated by the researcher: “(a) most teachers would find the intervention procedures suitable for the behaviour problem described, (b) the intervention was a fair way to handle the child's problem behaviour, (c) I liked the procedures used in this intervention, (d) I would suggest the use of this intervention to other teachers, and (e) the intervention did not result in negative side-effects for the child.” |
| Clarke & Duda (2019)  (USA) | Five-point scale completed by peer buddies pre- and post- intervention on modified quality of life indicators for the student related to social validation of the approach implemented, and direct observation data collected on “positive affect” of the student | - Social validation measures showed that intervals with “positive affect” for PE activities increased from *M*=8% (range 0– 27%) in baseline and withdrawal sessions to *M*=26% (range 4–33%) during intervention phases. - The mean peer buddies’ social validation measures demonstrated higher perception ratings after the intervention. (increased ratings: for “Friendships with her peers now are” from 4 to 4.6; for “Mia’s relationships with her teachers” from 3.3 to 4.2; for “general happiness” from 3.3 to 3.6; for “behavior is appropriate” from 2.8 to 3.4.') |
| Dunlap et al. (1995) (USA) | Six-point Likert rating scales (Dunlap, 1984) with 0-5 rating to assess happiness and interest of the student from videotaped sessions. | The intervention targeted meaningful outcomes (reduced challenging behaviours, increased on-task behaviour, increased happiness, and interest of student) |
| Flynn & Lo (2016) (USA) | Adapted version of Teacher Post-Intervention Acceptability and Importance of Effects Survey (Lane & Beebe-Frankenberger, 2004) with 11 statements and a Likert-type scale (1-5 rating) for rating trial-based Functional Analysis (TBFA) and intervention, and 2 additional open-ended questions on what teachers liked and what needed change completed by teachers | - All three teachers agreed (‘4’) or strongly agreed (‘5’) that TBFA and intervention procedures were easy to learn (*M* = 4.0), easy to perform in the classroom (*M* = 4.3, range 4.0–5.0), they would conduct TBFA (*M* = 4.7, range 4.0–5.0) and differential reinforcement of alternative behaviour (DRA) intervention with other students (*M* = 4.3, range 4.0–5.0), and would recommend TBFA (*M* = 4.0) and DRA (*M* = 4.7, range 4.0–5.0) to other teachers. - Regarding the outcomes, two of the teachers agreed or strongly agreed that the intervention implementation increased the students’ replacement behaviours (*M* = 3.7, range 3.0–4.0) and reduced their challenging behaviours (*M* = 3.7, range 3.0–4.0), while one teacher responded neither agreed nor disagreed (‘3’). - Responses to the open-ended questions showed that the training and feedback were very beneficial (Teachers 1 and 3), that “understanding behavioral function was useful” (Teacher 2), and that the implementation of the intervention produced positive outcomes (reduced medicine intake refusal for Student 1A and need for Student 3A to be placed in a more restrictive setting due to his ‘touching himself’ behaviour). |
|  |  | *(Continued)* |
| **Table S3.** *Detailed studies addressing RQ3* | | |
| Authors (Year) (Country) | Methods | Findings |
| Friedman & Luiselli (2008)  (USA) | Non-systematic social validity data obtained by staff reports. | - Staff reported that the intervention was acceptable, and they were pleased with how quickly the student responded. - Authors suggested that staff satisfaction could explain the continued use of the intervention after the study. |
| Lalli et al. (1993)  (USA) | Researchers mentioned social validity related data in the discussion section. | - The teachers taught the students socially acceptable behaviours (as an alternative to problem behaviours) that would help them acquire the reinforcers, targeting socially important goals. - The intervention had meaningful outcomes with more frequent interaction with the students throughout the day compared to prior to the study conditions. |
| Lane et al. (2006) (USA) | Social validity was assessed before and after the intervention by the teacher and assistant who completed the Intervention Rating Profile-15 (IRP-15; Martens, Witt, Elliott, & Darveaux, 1985), and by the student who completed a modified version of the Child Intervention Rating Profile (CIRP; Witt & Elliott, 1985). | - IRP-15 scores ranged from 15 to 90, and CIRP scores ranged from 7 to 42, with higher scores indicating higher treatment acceptability. - Teacher and assistant rated the intervention favourably (IRP-15: 74 and 80, respectively), with slightly increased ratings after intervention (75 and 86, respectively). - The student rated the intervention favourably (CIRP: 38 at both times; Witt & Elliott, 1985). |
| Larkin et al. (2016) (USA) | The Treatment Acceptability Rating Form (TARF) for intervention and modified version was used for the assessment procedures (Langthorne & McGill, 2011) were completed by teachers | - Teacher responses on the Treatment Acceptability Rating Form (TARF) demonstrated high acceptability for assessment and intervention procedures. - Teachers noted that they would be willing to use the procedures again and that they - Teachers noted that the procedures had a positive impact on the behaviour of students. |
| Moore et al. (2009)  (USA) | Anecdotal staff reports mentioned by researchers (no formal data collected) | - Anecdotal reports on the intervention and the involvement of teaching staff were positive. - Interventions were considered acceptable for the school environment, and useful and practical if oversight from the researchers would be absent. |
| Mueller & Kafka (2006)  (USA) | Teacher reported data (non-systematic social validity data) | Teacher reports regarding the social validity reflected positive perceptions about the effectiveness of the intervention and its use of use. Teachers also approved of the time requirements of the intervention. |
| (Mueller & Nkosi (2007)  (USA) | IRP-15 (Martens, et. al., 1985) completed by the teacher and paraprofessional after training had occurred | The IRP-15 results were 73 for the teacher and 78 for the paraprofessional of the student. These results demonstrate that the multicomponent intervention was considered as highly acceptable. |
|  |  | *(Continued)* |
| **Table S3.** *(Continued)* | | |
| Authors (Year) (Country) | Methods | Findings |
| Pennington et al. (2012) (USA) | Informal teacher reported data | The teacher reported that the procedures were easy to implement and that her positive feelings about the level of participation of the student increased. |
| Pitts et al. (2019)  (UK) | Questionnaire (5-point scale) on the social validity of intervention and training completed by 14 school staff (teachers and teaching assistants) | - Teaching staff reported that there was sufficient staff training about the strategies included in the Behaviour Support Plans and Individual Education Programs (*M*= 4.5, *SD* = 0.065). - The teaching staff agreed that the programs’ focus was on increasing the positive behaviour of students (*M*= 4.5, *SD* = 0.518) and that they targeted meaningful goals such as skills needed for the students (*M*= 4.5, *SD* = 0.650). - Staff felt comfortable implementing 1:1 sessions (*M*= 4.5, *SD* = 0.065), and most of them disagreed with the statement that Applied Behaviour Analysis induced pressure on classroom staff (*M*= 2.64, *SD* = 1.081). All participants agreed that the assessments were appropriate, and the lesson plans based on sufficient planning (*M*= 4.85, *SD* = 0.363), that there was regular monitoring (*M*= 4.78, *SD* = 0.425) and that the targets were adjusted when needed (*M*= 4.64, *SD* = 0.497). - All staff members agreed that there were benefits for the students from the interventions (*M* = 5, *SD* = 0.0). |

| **Table S4.** *Risk of Bias Critical Appraisal* | | | | | | | | | |
| --- | --- | --- | --- | --- | --- | --- | --- | --- | --- |
| Author (Year) (Country) | Risk of Bias (RoB) Tool (Reichow et al., 2018) | | | | | | | | |
|  | Selection Bias | | Performance Bias | | Detection Bias | | | | Other Bias Source |
|  | Sequence generation | Participant selection | Blinding (participant & personnel) | Procedural fidelity | Blinding outcome assessors | Selective outcome reporting | IOA | Data sampling | Other |
| Artman-Meeker et al. (2017) (USA) | Low | Low | ? | ? | ? | Low | Low | ? | High |
| Banda et al. (2009) (USA) | ? | Low | ? | Low | ? | Low | Low | ? | High |
| Banda et al. (2012) (USA) | ? | Low | ? | Low | ? | Low | Low | ? | High |
| Bethune & Wood (2013)  (USA) | Low | Low | ? | Low | High | Low | Low | ? | Low |
| Bloom et al. (2013) (USA) | ? | Low | ? | Low | High | Low | Low | Low | Low |
| Butler & Luiselli (2007)  (USA) | ? | Low | ? | High | ? | Low | Low | Low | Low |
| Calloway & Simpson (1998) (USA) | ? | Low | ? | High | ? | Low | Low | ? | Low |
| Cavalari et al. (2014) (USA) | ? | Low | ? | High | High | Low | High | ? | Low |
| Cihak & Gama (2008)  (USA) | Low | Low | ? | Low | High | Low | Low | Low | Low |
| Clarke & Duda (2019) (USA) | Low | Low | ? | Low | ? | Low | Low | ? | Low |
|  |  |  |  |  |  |  |  |  | *(Continued)* |

| **Table S4.** *(Continued)* | | | | | | | | | |
| --- | --- | --- | --- | --- | --- | --- | --- | --- | --- |
| Author (Year) (Country) | Risk of Bias (RoB) Tool (Reichow et al., 2018) | | | | | | | | |
|  | Selection Bias | | Performance Bias | | Detection Bias | | | | Other Bias Source |
|  | Sequence generation | Participant selection | Blinding (participant & personnel) | Procedural fidelity | Blinding outcome assessors | Selective outcome reporting | IOA | Data sampling | Other |

| Dunlap et al. (1995) (USA) | ? | Low | ? | High | ? | Low | Low | ? | High |
| --- | --- | --- | --- | --- | --- | --- | --- | --- | --- |

| Ellingson et al. (2000)  (USA) | Low | Low | ? | ? | High | Low | Low | ? | High |
| --- | --- | --- | --- | --- | --- | --- | --- | --- | --- |
| Flynn & Lo (2016)  (USA) | Low | Low | ? | Low | ? | Low | Low | ? | Low |
| Foran et al. (2015)  (UK) | ? | Low | ? | High | ? | Low | ? | ? | Low |
| Friedman & Luiselli (2008) (USA) | ? | Low | ? | High | High | Low | ? | Low | Low |
| Hansen & Wadsworth (2015) (USA) | ? | Low | ? | ? | ? | Low | Low | ? | Low |
| Karsh et al. (1995) (USA) | ? | Low | ? | High | ? | Low | Low | Low | Low |
| Lalli et al. (1993)  (USA) | ? | Low | ? | High | ? | Low | Low | ? | Low |
| Lane et al. (2006)  (USA) | ? | Low | ? | ? | ? | Low | Low | ? | High |

*(Continued)*

| **Table S4.** *(Continued)* | | | | | | | | | |
| --- | --- | --- | --- | --- | --- | --- | --- | --- | --- |
| Author (Year) (Country) | Risk of Bias (RoB) Tool (Reichow et al., 2018) | | | | | | | | |
|  | Selection Bias | | Performance Bias | | Detection Bias | | | | Other Bias Source |
|  | Sequence generation | Participant selection | Blinding (participant & personnel) | Procedural fidelity | Blinding outcome assessors | Selective outcome reporting | IOA | Data sampling | Other |

| Lang et al. (2010)  (USA) | ? | Low | ? | High | ? | Low | Low | Low | Low |
| --- | --- | --- | --- | --- | --- | --- | --- | --- | --- |

| Larkin et al. (2016) (USA) | Low | Low | ? | ? | ? | Low | Low | ? | Low |
| --- | --- | --- | --- | --- | --- | --- | --- | --- | --- |
| Lohrmann-O'Rourke & Yurman (2001) (USA) | ? | Low | ? | High | ? | Low | Low | ? | Low |
| Moore et al. (2009) (USA) | Low | Low | ? | High | ? | Low | ? | ? | Low |
| Mueller & Kafka (2006) (USA) | ? | Low | ? | Low | ? | Low | Low | ? | Low |
| Mueller & Nkosi (2007) (USA) | ? | Low | ? | High | ? | Low | ? | ? | Low |
| Myles & Hirsch (1996) (USA) | ? | Low | ? | High | High | Low | ? | Low | Low |
| Paris et al. (2019)  (UK) | ? | Low | ? | High | High | Low | ? | ? | Low |
| Pennington et al. (2012) (USA) | ? | Low | ? | High | ? | Low | Low | ? | Low |
| Rispoli et al. (2018) (USA) | ? | Low | ? | Low | ? | Low | Low | Low | Low |

| **Table S5.** *Quality Indicators* |  | | | | | | | |
| --- | --- | --- | --- | --- | --- | --- | --- | --- |
| Author (Date) | Quality Indicators (QI) (as found in Spear et al.,2013 paper based on Horner et al., 2005)  (Yes=quality indicator reported, No= quality indicator not reported) | | | | | | | |
|  | Quality Indicator 1 | | Quality Indicator 2 | Quality Indicator 3 | | Quality Indicator 4 | | |
|  | DV socially important | Society’s goals | Demonstration of functional relation | Cost- effective | Typical resources | Acceptable | Feasible | Continued use |
| Artman-Meeker et al. (2017) (USA) | Yes (on coaching) | Yes | Yes (Moderate) | No | Yes | Yes | Yes (on coaching) | No |
| Banda et al. (2009) (USA) | Yes | Yes | Yes (Moderate) | No | Yes | Yes | Yes | No |
| Banda et al. (2012) (USA) | Yes | Yes | Yes (Strong) | No | Yes | Yes | Yes | Yes |
| Bethune & Wood (2013) (USA) | Yes | Yes | Yes (Moderate) | Yes | Yes | Yes | Yes | Yes |
| Bloom et al. (2013)  (USA) | Yes | Yes | Yes (Strong) | No | Yes | No | No | No |
| Butler & Luiselli (2007) (USA) | Yes | Yes | Yes (Strong) | No | Yes | Yes | Yes | No |
| Calloway & Simpson (1998) (USA) | Yes | Yes | Yes (Moderate) | No | No | No | No | No |
| Cavalari et al. (2014) (USA) | Yes | Yes | Yes (Moderate) | No | Yes | No | No | No |
|  |  |  |  |  |  |  |  | *(Continued)* |
| **Table S5*.*** *(Continued)* |  | | | | | | | |
| Author (Date) | Quality Indicators (QI) (as found in Spear et al.,2013 paper based on Horner et al., 2005)  (Yes=quality indicator reported, No= quality indicator not reported) | | | | | | | |
|  | Quality Indicator 1 | | Quality Indicator 2 | Quality Indicator 3 | | Quality Indicator 4 | | |
|  | DV socially important | Society’s goals | Demonstration of functional relation | Cost- effective | Typical resources | Acceptable | Feasible | Continued use |
| Cihak & Gama (2008) (USA) | No | Yes | Yes (Moderate) | No | Yes | Yes | No | No |
| Clarke & Duda (2019) (USA) | No | Yes | Yes (Strong) | No | Yes | Yes | Yes | No |
| Dunlap et al. (1995) (USA) | Yes | Yes | Yes (Moderate) | No | Yes | No | No | No |
| Ellingson et al. (2000) (USA) | Yes | Yes | Yes (Moderate) | Yes | Yes | Yes | Yes | No |
| Flynn & Lo (2016) (USA) | Yes | Yes | Yes (Strong) | No | Yes | Yes | Yes | No |
| Foran et al. (2015) (UK) | Yes | Yes | Yes (Moderate) | Yes | Yes | No | No | No |
| Friedman & Luiselli (2008) (USA) | Yes | Yes | Yes (Strong) | No | Yes | Yes | No | Yes |
| Hansen & Wadsworth (2015) (USA) | Yes | Yes | Yes (Moderate) | No | Yes | No | Yes | No |
|  |  |  |  |  |  |  |  | *(Continued)* |

| **Table S5.** *(Continued)* |  | | | | | | | |
| --- | --- | --- | --- | --- | --- | --- | --- | --- |
| Author (Date) | Quality Indicators (QI) (as found in Spear et al.,2013 paper based on Horner et al., 2005)  (Yes=quality indicator reported, No= quality indicator not reported) | | | | | | | |
|  | Quality Indicator 1 | | Quality Indicator 2 | Quality Indicator 3 | | Quality Indicator 4 | | |
|  | DV socially important | Society’s goals | Demonstration of functional relation | Cost- effective | Typical resources | Acceptable | Feasible | Continued use |
| Karsh et al. (1995) (USA) | Yes | Yes | Yes (Strong) | No | Yes | No | Yes | No |
| Lalli et al. (1993) (USA) | Yes | Yes | Yes (Strong) | Yes | Yes | Yes | Yes | No |
| Lane et al. (2006) (USA) | Yes | Yes | Yes (Strong) | Yes | Yes | Yes | No | No |
| Lang et al. (2010) (USA) | Yes | Yes | Yes (Strong) | No | Yes | No | No | No |
| Larkin et al. (2016) (USA) | Yes | Yes | Yes (Moderate) | No | Yes | Yes | No | No |
| Lohrmann-O'Rourke & Yurman (2001) (USA) | Yes | Yes | Yes (Moderate) | No | Yes | No | No | No |
| Moore et al. (2009) (USA) | No | Yes | Yes (Moderate) | No | Yes | Yes | No | No |
|  |  |  |  |  |  |  |  | *(Continued)* |
| **Table S5.** *(Continued)* |  | | | | | | | |
| Author (Date) | Quality Indicators (QI) (as found in Spear et al.,2013 paper based on Horner et al., 2005)  (Yes=quality indicator reported, No= quality indicator not reported) | | | | | | | |
|  | Quality Indicator 1 | | Quality Indicator 2 | Quality Indicator 3 | | Quality Indicator 4 | | |
|  | DV socially important | Society’s goals | Demonstration of functional relation | Cost- effective | Typical resources | Acceptable | Feasible | Continued use |
| Mueller & Nkosi (2007) (USA) | Yes | Yes | Yes (Moderate) | No | Yes | Yes | No | No |
| Mueller & Kafka (2006) (USA) | Yes | Yes | Yes (Strong) | No | Yes | Yes | Yes | Yes |
| Myles & Hirsch (1996) (USA) | Yes | Yes | Yes (Strong) | No | Yes | No | No | No |
| Paris et al. (2019) (UK) | Yes | Yes | Yes (Moderate) | No | Yes | No | No | Yes |
| Pennington et al. (2012) (USA) | Yes | Yes | Yes (Moderate) | No | Yes | No | Yes | No |
| Pitts et al. (2019) (UK) | No | Yes | Yes (Strong) | No | Yes | Yes | Yes | No |
| Rispoli et al. (2018) (USA) | Yes | Yes | Yes (Strong) | No | Yes | No | No | No |
|  |  |  |  |  |  |  |  |  |
